# Modest enhancements to conventional grassland diversity improve the provision of pollination services

**DOI:** 10.1111/1365-2664.12608

**Published:** 2016-02-09

**Authors:** Katherine A. Orford, Phil J. Murray, Ian P. Vaughan, Jane Memmott

**Affiliations:** ^1^ School of Biological Sciences University of Bristol Bristol Life Sciences Building 24 Tyndall Avenue Bristol BS8 1TQ UK; ^2^ Rothamsted Research North Wyke Okehampton Devon EX20 2SB UK; ^3^ Cardiff School of Biosciences Cardiff University The Sir Martin Evans Building Museum Avenue Cardiff CF10 3AX UK

**Keywords:** agro‐ecosystems, crop yields, ecosystem services, functional diversity, grassland diversity, plant richness, pollinators, visitation networks

## Abstract

1. Grassland for livestock production is a major form of land use throughout Europe and its intensive management threatens biodiversity and ecosystem functioning in agricultural landscapes. Modest increases to conventional grassland biodiversity could have considerable positive impacts on the provision of ecosystem services, such as pollination, to surrounding habitats.
2. Using a field‐scale experiment in which grassland seed mixes and sward management were manipulated, complemented by surveys on working farms and phytometer experiments, the impact of conventional grassland diversity and management on the functional diversity and ecosystem service provision of pollinator communities were investigated.
3. Increasing plant richness, by the addition of both legumes and forbs, was associated with significant enhancements in the functional diversity of grassland pollinator communities. This was associated with increased temporal stability of flower–visitor interactions at the community level. Visitation networks revealed pasture species *Taraxacum* sp. (*Wigg*.) (dandelion) and *Cirsium arvense* (*Scop*.) (creeping thistle) to have the highest pollinator visitation frequency and richness. *Cichorium intybus (L.)* (chichory) was highlighted as an important species having both high pollinator visitation and desirable agronomic properties.
4. Increased sward richness was associated with an increase in the pollination of two phytometer species; *Fragaria* × *ananassa* (strawberry) and *Silene dioica* (red campion), but not *Vicia faba* (broad bean). Enhanced functional diversity, richness and abundance of the pollinator communities associated with more diverse neighbouring pastures were found to be potential mechanisms for improved pollination.
5. *Synthesis and applications*. A modest increase in conventional grassland plant diversity with legumes and forbs, achievable with the expertise and resources available to most grassland farmers, could enhance pollinator functional diversity, richness and abundance. Moreover, our results suggest that this could improve pollination services and consequently surrounding crop yields (e.g. strawberry) and wildflower reproduction in agro‐ecosystems.

## Abstract 2

A modest increase in conventional grassland plant diversity with legumes and forbs, achievable with the expertise and resources available to most grassland farmers, could enhance pollinator functional diversity, richness and abundance. Moreover, our results suggest that this could improve pollination services and consequently surrounding crop yields (e.g. strawberry) and wildflower reproduction in agro‐ecosystems.

## Introduction

Understanding how species' interactions affect ecological function is central to conservation biology. For sustainable land management, land managers can engineer community composition through intertrophic relationships to enhance ecosystem services. Examples include providing food for pollinators (Potts *et al*. 2003) to enhance crop pollination and providing alternative prey for predatory insects (Symondson, Sunderland & Greenstone 2002) which provide pest control. Manipulating basal trophic levels has been shown to have significant bottom‐up effects on higher trophic‐level diversity and ecosystem functioning (Novotny *et al*. 2006; Haddad *et al*. 2009; Scherber *et al*. 2010). A diverse plant community provides opportunities for niche diversification and coexistence of associated species (Novotny *et al*. 2006; Rzanny & Voigt 2012), with a diversity of functional traits (Hooper *et al*. 2005) which has been found to improve ecosystem service provision (Albrecht *et al*. 2012). This positive relationship between functional diversity and ecosystem service provision is associated with complementary niche partitioning between functional groups which can enhance the temporal and spatial stability of ecosystem processes (Naeem & Li 1997; Ebeling *et al*. 2008; Macfadyen *et al*. 2011; Brittain, Kremen & Klein 2013). This is true for the stability of pollination services; if complementary pollinator functional groups visit different plant species, or the same plant species at different times, this can enhance the overall visitation and pollination of plant communities (Hoehn *et al*. 2008; Albrecht *et al*. 2012; Brittain, Kremen & Klein 2013). Functional facilitation can also occur, for example interactions between pollinators may force individuals to move from plant to plant facilitating cross‐pollination (Greenleaf & Kremen 2006). Furthermore, communities with high functional diversity are more likely to include functionally effective individuals or groups (Albrecht *et al*. 2012). Although a number of hypotheses explain such cascading ecosystem‐level processes (Hooper *et al*. 2005), much of the work has been theoretical and the putative causal factors rarely manipulated in the field at the community scale.

In this study, conventional grasslands used for livestock production provide a model system to determine how manipulation of basal trophic levels (by modest increases in sward richness and concomitant cutting and grazing treatments) affects pollination. Few studies have focussed on ecosystem service provision by conventional grasslands (Potts *et al*. 2009; Power & Stout 2011). Moreover, grassland agri‐environment schemes have had limited effect in diversifying these homogeneous habitats to enhance pollination (Kleijn & Sutherland 2003; Scheper *et al*. 2013). Whilst it is unrealistic to restore managed grasslands to their former high diversity, as they are a product of low‐intensity farming systems (van Dijk 1991), modest changes to grassland biodiversity via agri‐environment schemes could have extensive benefits due to its widespread cover [grasslands covers 30–40% of European agricultural areas (Sokolović, Radović & Tomić 2011)]. Moreover, spillover of pollinators from grasslands to surrounding habitats could enhance pollination at the landscape scale (Klein, Steffan‐Dewenter & Tscharntke 2003; Kremen *et al*. 2004).

There are three objectives to our study: (i) to determine the impact of grassland plant richness and management (cutting and grazing) on pollinator functional diversity and the consequence of functional diversity on the temporal stability of community flower–visitor interactions over the season; (ii) to determine which grassland plant species provide disproportionate support to pollinators in terms of the number and richness of visitors, thus providing target species for restoration projects; and (iii) to determine whether increased pasture plant richness is associated with enhanced pollination services as measured by seed/fruit set, weight and quality of three phytometer species. In the context of these objectives, functional diversity is defined as ‘measuring functional trait diversity, where functional traits are components of an organism's phenotype that influence ecosystem‐level processes' (Petchey & Gaston 2006).

## Materials and methods

Three approaches were used as follows: a field experiment with replicate treatment plots; a correlative approach, which used a pre‐existing gradient of pasture plant diversity on multiple farms; and a phytometer approach whereby three plant species were placed adjacent to pastures to assay pollination spillover.

### The Field Experiment

We assessed the impact of manipulating conventional grassland sward diversity and management on pollinator communities using a replicated field‐scale experiment from May to September 2011. This was carried out at Rothamsted Research, North Wyke, Devon, UK (50°46′N 3°54′W). A split‐plot design was used with four replicate blocks to investigate the effect of two treatments: sward diversity and sward management (Figs S1 and S2 in Supporting information). Two plot sizes were used 0·1 ha (grazed plots) and 0·07 ha (cut plots).

Sward diversity was manipulated by sowing three seed mixes: grass only, grass–legume and grass–legume–forb (Tables S1 and S2 for species lists). Species were selected from a review of the biodiversity and agronomic value of grassland species by Mortimer *et al*. (2006) as potential target species for agri‐environment scheme seed mixes. Each sward diversity treatment was split into two subplots which were subjected to one of two management regimes: (i) grazing: grazing by cattle from April/May to early June, no grazing from early June to August, and moderate grazing by cattle from August to October (two animals per 0·1‐ha plot); (ii) cutting: cut early June, grazing by cattle from late August to October.

#### Sampling of pollinators and flowering plants

Plant and pollinator surveys were carried out within a 500‐m^2^ sampling area in the centre of each plot by zigzag walking for 25 min catching all insects observed on flowers. Each plant–pollinator ‘visitation interaction’ was recorded (Table S3) by identifying the plant species in the field and collecting the visitor for later identification by taxonomists. Flower‐visiting Hymenoptera, Lepidoptera, Coleoptera and Diptera were collected; all four orders carry pollen (Orford, Vaughan & Memmott 2015). All plots within a block [six subplots including all treatment combinations (Figs S1 and S2)] were sampled in a random order per day (09:00–17:00 h) during warm, dry conditions. Between May and September, each of the four blocks was sampled 24 times; each sward type 192 times and management type 288 times (Fig. S1). Following each survey, the number of floral units of each plant species was counted along a 25 × 2‐m transect in each plot.

### Farm Surveys

To increase the spatial scale of our study, and to measure pollinator population‐level responses, we investigated the effect of pasture plant species richness across ten independent farms (separated by at least 6·5 km) across south‐west England, scattered north and south of Bristol and Bath (map Fig. S3). All were mixed farms, with arable crops and pasture. The farms were selected as they were used in a previous study (Macfadyen *et al*. 2009), and therefore, data on their management and pasture plant diversity were available. Two pasture fields per farm (20 fields in total) were selected based on their plant species richness to cover a gradient of diversity. Richness was measured using two 30 × 2‐m transects where 1–10 species = ‘low’ diversity, 11–20 species = ‘intermediate’ diversity and 21–30 species = ‘high’ diversity. Abiotic factors and surrounding landscape features, as well as differing management, are potential causes of each pastures' plant richness. The pastures had similar management; all were grazed by cattle, used herbicides to spot‐spray undesirable species, including *Rumex, Cirsium* and *Senecio* sp., and used nitrogen‐based fertilizers. All fields had hedgerows, and field sizes are included in Table S4. Whilst detailed information on the surrounding landscape was not recorded, we know it is dominated by agricultural land with some woodland cover. Data on local pesticide use were not available; however, none of the farms were organic so pesticides are likely to have been used. Plant–pollinator surveys were carried out on each pasture five times between May and August 2012, following the same survey protocol as the field experiment (i.e. zigzag walking within a central 500‐m^2^ area for 25 min followed by 30 × 2‐m^2^ transects to identify the plant species and count floral units).

### Phytometer Experiment

Plant phytometers were used to bioassay the pollination service of the pasture on each of the ten farms. During their flowering period, five individuals of each phytometer species, strawberry variety ‘Symphony’, *Fragaria* × *ananassa*, (Duch.): Rosaceae; broad bean variety ‘Sutton’, *Vicia faba* (L.): Fabaceae and red campion *Silene dioica* ((L.) Clairv.): Caryophyllaceae*,* were positioned at the margin of one pasture field in each farm in early June 2013. As red campion is dioecious, five male plants were put out on the farms in addition to the five female plants. Prior to the experiment, the strawberry and bean plants were grown from seed in greenhouses until flowering and red campion plants, which were approximately 3 years old, were stored in polytunnels. Phytometers were relocated to the farms whilst in bud.

Strawberry is a crop commonly grown in the UK. The plants are self‐compatible and whilst both wind and self‐pollination occur, cross‐fertilization is favoured (Free 1993). A wide diversity of insects visit strawberry flowers due to their open structure (Dimou *et al*. 2008; Klatt 2013). Strawberries have increased weight and fewer deformities if insect‐pollinated (Chagnon, Gingras & Deoliveira 1989; Free 1993; Dimou *et al*. 2008; Klatt 2013). Broad bean is another crop commonly grown in the UK. It has partial cross‐fertilization with significantly higher seed numbers and weight when insect‐pollinated (Free 1966; Aouar‐Sadli, Louadi & Doumandji 2008). Broad bean has closed papilionaceous flowers that only pollinators with long and strong mouthparts can access, predominantly bees (Free 1966, 1993; Aouar‐Sadli, Louadi & Doumandji 2008). Red campion is a wildflower present in hedgerows and woodlands in the study region. It is dioecious and requires insect pollination and seed‐set is related to the amount and identity of pollen deposited on the stigmas (Montgomery, Soper & Delph 2010). It is pollinated by insects with long mouthparts including bees, hoverflies and butterflies (Charlton 2013).

Location of the phytometer plants was prioritized to ensure that surrounding features were similar between farms, for example hedgerows and tree cover. Plants were put in areas where disturbance by cattle/tractors was minimal. Wild specimens of the phytometer species were not found in close proximity. Chicken wire fences protected the plants from grazing animals, and the phytometers were left in the field for 2 weeks to allow pollination. Four plant–pollinator surveys were carried out in the centre of the associated pasture field during this period following the same protocol as the plot experiment surveys where a zigzag walk was carried out within a 500‐m^2^ area over 25 min and associated 30 × 2‐m^2^ plant transect surveys.

In late June, the phytometer plants were collected from the farms and kept in enclosed polytunnels to allow fruits to mature. Any new flower buds were removed. Strawberry fruits were picked when ripe and weighed. Mean fruit weight was calculated per farm. Each fruit was classed based on commercial deformity grades (European Commission (2007)) where fruits without or with slight aberrations were sorted into Class 1, whereas severe aberrations lead to Class 2 classification. Broad bean seed pods were collected at maturity, and the seeds were counted and weighed; mean seed count (per pod) and seed weight were calculated per farm. Bean plants were still young when harvested. Seed capsules of red campion were collected, the number of seeds per capsule was counted, and a mean was calculated per farm.

### Analysis

#### Objective 1: The impact of plant richness and grassland management on pollinator functional diversity and the resulting temporal stability of flower visitation

Pollinator functional diversity of each plot of the field experiment and each field of the farm surveys was calculated. Functional diversity was based on the feeding niche of the pollinator species recorded, which we based on the plant families that each species is known to visit. This was to ascertain the potential complementarity of diets within the pollinator communities. The feeding niche of each pollinator species recorded was determined from the interactions recorded in the current study and by a literature search. This established the pollinator community's potential visitation to plants not just within the grassland but to surrounding habitats. The search was carried out using ISI Web of Knowledge, the BSBI data base and English Nature reports and included studies from 1883 to 2010. This added 2398 flower–insect interactions to the 143 interactions from the field experiment data and 2189 interactions to the 84 interactions observed in the farm surveys.

Using these interaction data, a functional dendrogram was created in r (R Core Development Team 2012) for the field experiment and another for the farm surveys by calculating pairwise distances between pollinator species and then using a clustering algorithm (Petchey & Gaston 2007). This was based on similarities in feeding niche of the pollinators (plant families had a binary score; either visited or not visited by the pollinator species) to describe the functional relationships between the pollinator species recorded. Functional diversity was calculated for each plot/field as the total branch length of the functional dendrogram between all the species sampled (Petchey & Gaston 2007) using the ‘jaccard index’ (ade 4 package) (Dray & Dufour 2007) and ‘treedive’ (vegan package) (Oksanen *et al*. 2012) functions. Values for functional diversity do not have a directly interpreted meaning but provide a means of comparison; the higher the functional diversity the greater the complementarity in feeding niches of the pollinator community and the lower the redundancy. Pollinator species richness (count of species) and abundance (count of individuals) of the plots/fields were calculated to test whether differences in functional diversity were distinct from differences in pollinator species richness or abundance.

The coefficient of variation of visitation (CV) was used as a measure of the temporal variability in the visitation interactions between all plants and pollinators surveyed over the entire sampling season to determine the temporal stability of the potential ecosystem service (adapted from Macfadyen *et al*. (2011)). For the field experiment, the data set was separated into six time periods. The CV per plot was calculated across the six time periods as the standard deviation in number of visitation interactions divided by the mean number of visitation interactions. For the farm surveys, the CV was calculated for each field with the data set split into five sampling periods.

To compare pollinator functional diversity, pollinator species richness, pollinator abundance (response variables) between plots of the field experiment, general linear mixed‐effects models were used [GLMM; ‘lme4′ in r (Bates, Maechler & Bolker 2012)]. Plot treatments ‘sward type’ and ‘management’ (and their interaction) were fixed factors. ‘Sward type’ and ‘block’ were treated as nested random factors to account for the arrangement of the plots. Models were compared with maximum likelihood ratio tests, following model simplification, to evaluate the significance of the predictors on the response variable (Zuur *et al*. 2009). *Post hoc* Tukey tests (Hothorn, Bretz & Westfall 2008) were used to determine where differences in the response variable lay between sward types (package ‘multcomp’ Hothorn, Bretz & Westfall 2008). Plots of the residuals were used to check the fits of the models. A GLMM with the same random effects structure was used to test the relationship between CV (response) and pollinator functional diversity (predictor) within the plots. For both the field experiment and farm surveys, correlation coefficients were calculated between pollinator functional diversity, species richness and abundance to assess the degree of colinearity.

To determine the relationship between pollinator functional diversity, pollinator species richness, pollinator abundance (response variables) and plant species richness in the farm surveys, GLMMs were also fitted. Plant species richness was treated as a fixed effect and farm was treated as a random factor (to account for abiotic and management differences). Subsequent models were fitted omitting the predictor variable (intercept‐only model). The two models were compared with a likelihood ratio test. This method was also used to test the relationship between CV (response) and pollinator functional diversity (predictor) within the fields.

#### Objective 2: Which grassland plant species have disproportionately positive effects upon pollinator abundance and diversity?

A quantitative plant–pollinator visitation network of the interactions recorded was created for both the field experiment and farm surveys. Following Hegland *et al*. (2010), we consider the functional value of a species to depend on its interaction frequency and interaction richness (the number of visitors and the number of visitor species, respectively); the greater the interaction frequency and richness, the more functionally valuable the plant species. Floral abundance was accounted for by dividing the number of interactions by the number of floral units of each species.

#### Objective 3: The impact of pasture plant species richness on the pollination of crop and wildflower species

Linear regression was used to test for relationships between pasture plant species richness and the phytometer response variables; strawberry fruit weight (mean fruit weight per farm) and deformity (the proportion of Class 1 fruits per farm, arcsine‐square‐root transformed), mean broad bean seed count (per pod) and weight, per farm. A generalized linear model (GLM) with Poisson errors assessed the relationship between plant species richness and seed count (mean seed count per capsule per farm) of red campion. Pollinator functional diversity was calculated for the 2013 pollinator surveys. Linear regression tested the relationship between plant species richness and pollinator functional diversity.

Three alternative predictor variables – pollinator functional diversity, abundance and species richness (associated with the neighbouring pasture) – were investigated as potential mechanisms behind any increased pollination of the strawberry phytometers (response variables listed above), using linear regression. A GLM with Poisson errors was used in association with red campion seed count. The differences in Akaike Information Criterion (AIC) values were calculated as a means of comparing the three alternative models for each phytometer response variable (Burnham & Anderson 2004).

## Results

### Objective 1: The Impact of Sward Diversity and Management on Pollinator Functional Diversity and the Resulting Temporal Stability of Flower Visitation

In the field experiment, 4169 flower visitors were collected comprising 166 insect species: 12 bee species, 34 hoverfly species, 90 non‐hoverfly Diptera species, 18 Coleoptera species and 2 Lepidoptera species (Table S5). In the 2012 farm surveys, a gradient of 9–36 plant species per 60 m^2^ was recorded and 1530 flower visitors were collected, comprising 146 insect species: 15 bee species, 15 hoverfly species, 76 non‐hoverfly Diptera species and 23 Coleoptera species (Table S6).

**Figure 1:**
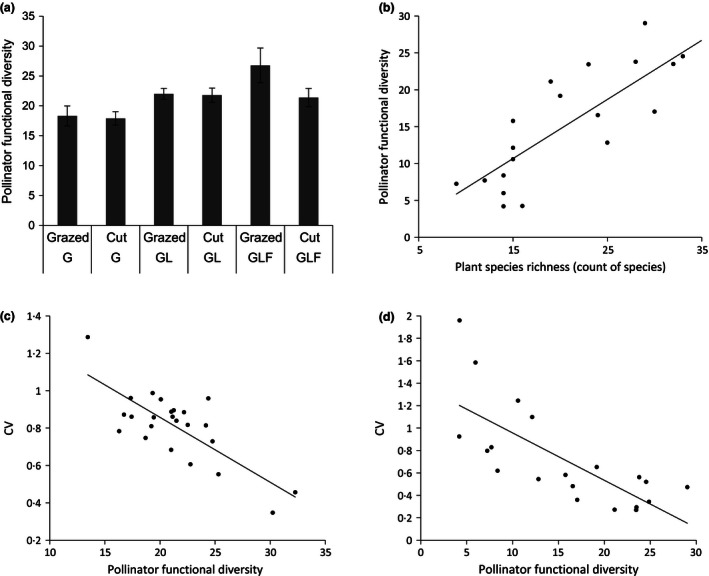
(a) Pollinator functional diversity in the field experiment: G = grass; GL = grass–legume; GLF = grass–legume–forb; error bars represent standard error. (b) Pollinator functional diversity and plant species richness (per 30 × 2‐m transects) within the fields of the farm surveys. (c) The relationship between pollinator functional diversity and the temporal stability in flower visitation [the coefficient of variation in visitation (CV)] for the field experiment and (d) farm surveys.

Pollinator functional diversity significantly increased as sward diversity increased in the field experiment (χ^2^ = 125·57, d.f. = 1, *P *=* *0·0052) (Fig. 1a). The difference was between grass only and grass–legume–forb plots (*z* = 3·61, d.f. = 7 *P *< 0·001) (Fig. 1a). There was no significant difference in pollinator functional diversity between cut and grazed management (χ^2^ = 123·22, d.f. = 1, *P *=* *0·13). No significant difference in pollinator species richness was found between sward types (χ^2^ = 155·97, d.f. = 1, *P *=* *0·069) or management type (χ^2^ = 153·25, d.f. = 1, *P *=* *0·099). Sward type did have a significant effect on pollinator abundance (χ^2^ = 264·24, d.f. = 1, *P *=* *0·026) being higher in grass–legume–forb plots than grass only plots (*z* = 2·98, d.f. = 7, *P *=* *0·0081). Management type did not have any significant effect on pollinator abundance (χ^2^ = 261·01, d.f. = 1, *P *=* *0·072).

In the farm surveys, pollinator functional diversity was significantly positively associated with plant species richness (χ^2^ = 14·542, d.f. = 1, *P *< 0·001) (Fig. 1b) as was pollinator species richness (χ^2^ = 10·831, d.f. = 1, *P *<* *0·001) and pollinator abundance (χ^2^ = 9·178, d.f. = 1, *P *=* *0·002).

In both the field experiment and farm surveys, the response variables, pollinator functional diversity, species richness and abundance, were collinear (*r *≥* *0·77; Table S7). However, they responded differently to some of the treatments (e.g. pollinator functional diversity c.f. pollinator species richness) and so are considered separately.

It could be argued that high pasture plant species richness indicates management that is generally ‘sympathetic’ to biodiversity across the farm and so pollinator communities could have been responding to farm‐scale rather than field‐scale management. We used a Wilcoxon matched‐pairs signed‐ranks test where fields were paired per farm to test whether the fields differed in pollinator functional diversity, thereby removing the farm effect. There was a significant difference between fields of the same farm (*V* = 50, *n* = 10, *P *=* *0·02); hence, variation in pollinator communities was not due to farm‐scale management but to individual fields.

A significant linear negative relationship existed between the functional diversity of the pollinator community and the temporal variability of insect–flower visitation in both the field experiment (χ^2^ = 21·70, d.f. = 1, *P *<* *0·001) (Fig. 1c) and farm surveys (χ^2^ = 11·86, d.f. = 1, *P *<* *0·001) (Fig. 1d). Thus, as pollinator functional diversity increases, the temporal stability of flower visitation increases.

### Objective 2: Which Grassland Plant Species have Disproportionately Positive Effects on Pollinator Abundance and Diversity?

In the field experiment, *Taraxacum* sp. (F. H. Wigg.) was the most important species for supporting pollinators (per floral unit), attracting 35% of all pollinator visits and 33% of all pollinator species, followed by *Ranunculus acris* (L.) and *Cardamine pratensis* (L.). Surprisingly, grass species including *Alopecurus pratensis* (L.) and *Dactylis glomerata* (L.) were commonly visited by pollinators (Fig. S4a, Table S3), predominantly by Diptera within the Syrphidae and Muscoidea families. To confirm that the insects were feeding on the pollen and verify the grasses as a protein source, a stratified random sample of 60 individuals of 23 of the Diptera species caught on the grasses in the field experiment were dissected. In 72% of cases, Poaceae pollen was present in the abdomen, suggesting the dietary importance of grasses to Diptera.

In the farm surveys, *Cirsium arvense* ((L.) Scop.) was found to be the most important species, attracting 17% of all pollinator visits and 18% of all pollinator species followed by *Cirsium palustre* ((L.) Scop.) and *Crepis capillaris* ((L.) Wallr.) (Fig. S4b)*. Taraxacum* sp. floral units accounted for 0·02% of all floral units within the plots and *C. arvense* floral units accounted for 0·08% of floral units in the farm surveys. Therefore, the results are not necessarily a consequence of these species' abundance.

### Objective 3: The Impact of Pasture Plant Species Richness on the Pollination of Crop and Wildflower Species

In the 2013, pasture surveys (associated with the phytometer experiment), 349 insects were collected comprising 72 insect species: 9 bee species, 17 hoverfly species, 36 non‐syrphid Diptera species, 5 Coleoptera species and 3 Lepidoptera species. A gradient of 9–28 plant species per field was recorded per transect (30 × 2‐m). With regard to seed/fruit production of the phytometer plants, 161 strawberries were harvested, 136 broad bean seeds were collected from 44 pods, and 39 280 red campion seeds were collected from 274 seed capsules.

**Figure 2:**
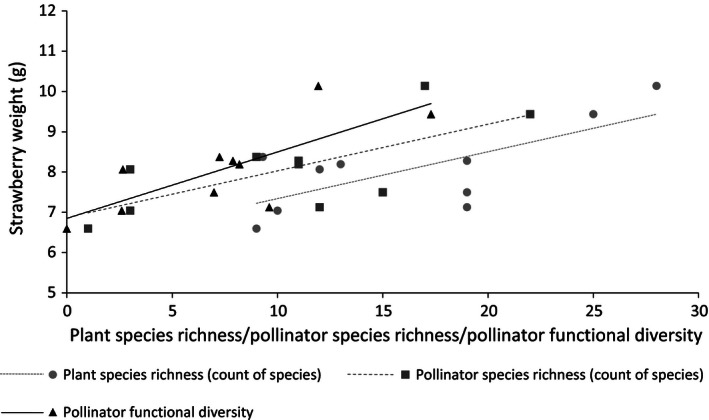
The relationship of plant species richness, pollinator species richness and pollinator functional diversity of the 10 neighbouring pastures with the mean fruit weight of the strawberry phytometers.

**Figure 3:**
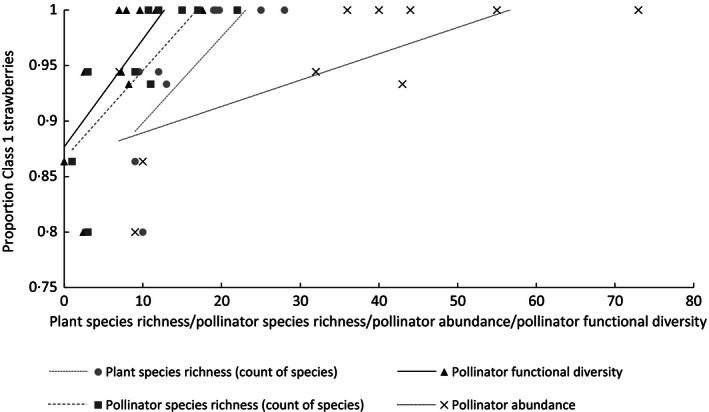
The linear relationship of plant species richness, pollinator species richness, pollinator abundance and pollinator functional diversity of the 10 neighbouring pastures with the proportion of Class 1 strawberry fruits of the phytometers.

**Figure 4:**
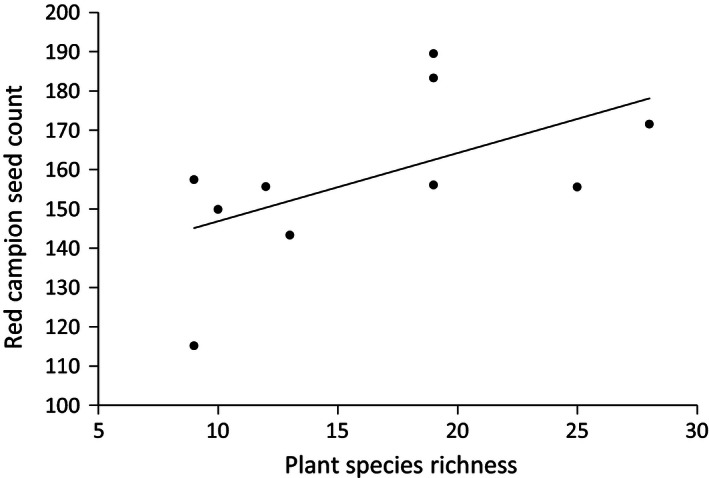
The relationship between pasture plant species richness and mean seed count of red campion phytometer specimens across 10 farms.

Pasture plant species richness was significantly and positively associated with strawberry fruit weight (*t* = 2·86, d.f. = 9, *P *=* *0·021, Fig. 2) and proportion of Class 1 fruits (*t* = 4·62, d.f. = 9, *P *=* *0·002, Fig. 3). Plant species richness was also significantly positively associated with seed count per capsule of red campion (*z* = 2·79, d.f. = 9, *P *=* *0·005, Fig. 4). For broad bean, there was no significant relationship detected between pasture plant species richness and seed count per pod (*t* = −1·28, d.f. = 8, *P *=* *0·24) or seed weight (*t* = −1·43, d.f. = 1,7, *P *=* *0·20). There was a significant positive relationship between pasture plant species richness and pollinator functional diversity (*t* = 4·031, d.f. = 1,8, *P *=* *0·004) as in Objective 1. Pollinator functional diversity, richness and abundance were all responsible for enhanced pollination of the phytometer plants to varying extents (Table 1, Figs 2 and 3).

**Table 1:** The results of the linear models (response variables: strawberry weight and class) and glms (response variable: red campion seed count). ‘*t*’ is reported for the linear models and ‘*z*’ for the glms. Broad bean is not included here as there was no significant relationship detected between the grassland diversity and its pollination

Phytometer measurement (response)	Pollinator community parameter (predictor)	*t*/*z*	*P*	ΔAkaike Information Criterion
Strawberry weight	Functional diversity	*t* = 3·25	0·012^a^	0
	Species richness	*t* = 2·84	0·022^a^	1·44
	Abundance	*t* = 1·66	0·14	5·47
Strawberry class	Functional diversity	*t* = 3·2	0·013^a^	0
	Species richness	*t* = 4·14	0·0032^a^	3·25
	Abundance	*t* = 3·43	0·009^a^	0·81
Red campion seed count	Functional diversity	*z* = 0·47	0·64	66·29
	Species richness	*z* = 1·3	0·19	0
	Abundance	*z* = 1·04	0·3	65·43

a Denotes a significant result.

## Discussion

We found modest increases in conventional grassland plant species richness to be associated with significantly enhanced pollination services, potentially enhancing crop yields and wildflower reproduction in adjacent habitats. In what follows, we discuss our findings in relation to our original objectives and end by considering practical management options for grassland management in the light of our results.

### Can Higher Sward Diversity Enhance Ecosystem Functioning and Services?

In the field experiment, both legumes and forbs were needed to create a suitable ecological infrastructure to enhance pollinator functional diversity. Sward richness in the farm surveys was also positively associated with pollinator functional diversity. Complementarity in resource use of the more functionally diverse pollinator communities is a potential mechanism behind the lower temporal variability in flower visitation found at both scales. This has potential implications for a more temporally stable ecosystem service. The fact that relationships found in the field experiment held true in the farm‐scale studies, where population responses were measured, supports the use of small‐scale experiments with pollinators.

Increased pollinator functional diversity, species richness and abundance were associated with increased pollination of strawberry. As these pollinator community variables were correlated, it is difficult to determine the causative factor behind enhanced pollination. However, AIC values suggested pollinator functional diversity and richness to be equally effective in increasing strawberry weight, whilst richness appeared to have the biggest positive effect on strawberry quality (class) followed by abundance and functional diversity. Spatial complementarity of pollen deposition has been highlighted as a mechanism behind increased strawberry pollination and resulting quality; large and average‐sized bees pollinate the apical stigmata, and small‐sized bees pollinate the basal stigmata (Chagnon, Gingras & Deoliveira 1993). Maximizing fruit weight and quality will achieve the highest prices for growers providing an incentive to encourage these natural ecosystem processes. However, given we use phytometers, the results are not directly related to estimates of farm‐scale crop production.

The seed‐set of red campion was positively associated with sward richness; this however could not be explained by pollinator functional or species diversity or abundance. The tubular flower structure of red campion is likely to lead to a more specialized pollination syndrome than strawberry. Therefore, the diversification of pollinator feeding niches may be redundant. Although many studies focus on the value of natural systems in providing benefits to managed systems, few have considered the value of managed systems in maintaining wildflower pollination (Blitzer *et al*. 2012).

Pasture plant species richness was not associated with improved pollination of broad bean. The flower of broad bean is even more specialized than red campion and is predominantly pollinated by large bees (Free 1966; Aouar‐Sadli, Louadi & Doumandji 2008) and so the issue of redundancy is raised again. It is likely that large bees forage at a scale greater than that of individual pastures and consequently the local effect of increased botanical richness may not translate into enhanced pollination.

An unexpected outcome of the field experiment was that the pollinator community parameters did not significantly differ between the cut and grazed treatments. One possible reason could be that surrounding landscape features provided a refuge for the pollinators during cutting. The realized plant species composition of the plots (Table S2) show there is not a great difference in the species richness between the cut and grazed plots.

It is important to note that a limitation of our work was that we utilized a pre‐existing gradient of pasture species richness in the farm surveys and therefore a correlative approach; manipulative experiments at the farm scale are needed to really prove the relationship between plant richness and pollination.

### Which Species should be Introduced into Seed Mixes?

*Taraxacum* sp. and *C. arvense* were the most valuable floral resources to pollinators in the visitation networks of the field experiment and farm data, respectively. It could be that these species have a high visual appearance to pollinators due to their large flowers. Unfortunately, these species have little agronomic value and may even be detrimental (Mortimer *et al*. 2006). This trade‐off between agronomic and biodiversity benefits must be considered in agri‐environment schemes and species that provide benefits to the farmer as well as the environment must be identified. We highlight *Cichorium intybus* a species sown into the experimental plots, as a possible target species. It had high visitation providing resources for pollinators and also agronomic value; it has antihelminthic properties which result in increased weight gain in lambs (Marley *et al*. 2006) and a deep tap root that captures fertilizers (Moore, Sanford & Wiley 2006).

### Conclusion

Without widespread changes in the management of improved grasslands, the decline of many pollinator species is likely to continue (Tscharntke *et al*. 2005; Carvell *et al*. 2006). Manning *et al*. (2015) show that increasing the diversity of grassland plants is likely to be associated with increases in the diversity of a wide range of taxa, with possible conservation and ecosystem service benefits. Our work adds a new aspect to this evidence by demonstrating positive effects on pollination services. Techniques to improve pasture plant species richness are achievable with the expertise and resources available to most grassland farmers. These can include sowing desirable seed mixes, spreading green hay cut from species‐rich sites, sward disturbance (e.g. turf removal, harrowing or use of herbicides), sowing hemiparasitic species, for example *Rhinanthus minor* and reducing phosphorous and potassium levels (Pywell *et al*. 2012). A desirable balance between agronomic performance of the grassland and its diversity must be considered when choosing such management options. The cascading bottom‐up effects of plants, seen at two spatial scales here, demonstrate that modest increases in grassland floral richness is an option for land managers wanting to improve the value of their land for pollinators and ultimately enhance pollination in agricultural habitats.

## Data accessibility

All data (pollinator surveys, plant surveys and phytometer experiment) are available from the Dryad Digital Repository: doi:10.5061/dryad.tp0d0 (Orford *et al*. 2016).

## Supporting information


**Fig. S1**. Experimental design of one of the four replicate blocks of the field experiment.
**Fig. S2.** Layout of the field experiment.
**Fig. S3.** Locations of the 10 farms.
**Fig. S4.** Grassland plant species' interaction frequency and interaction richness.
**Table S1.** Plant species of the seed mixes of the field experiment.
**Table S2.** Realized composition of the three sward types under grazing or cutting management of the field experiment.
**Table S3.** Plant species and their insect visitor species.
**Table S4.** Field sizes.
**Table S5.** Pollinator species lists from the different sward types of the field experiment.
**Table S6.** Species lists of pollinators found on each farm (2012 and 2013 surveys).
**Table S7.** Correlations between the parameters of the pollinator communities in both the field experiment and farm pollinator surveys.
