# Supplementary material for: Modest enhancements to conventional grassland diversity improve the provision of pollination services
Source: J Appl Ecol. 2016 Feb 9;53(3):906–15. doi: 10.1111/1365-2664.12608 (PMC4996327; doi:10.1111/1365-2664.12608)
Supplement: Supplementary file 1 — Fig. S1. Experimental design of one of the four replicate blocks of the field experiment. Fig. S2. Layout of the field experiment. Fig. S3. Locations of the 10 farms. Fig. S4. Grassland plant species' interaction frequency and interaction richness. Table S1. Plant species of the seed mixes of the field experiment. Table S2. Realized composition of the three sward types under grazing or cutting management of the field experiment. Table S3. Plant species and their insect visitor species. Table S4. Field sizes. Table S5. Pollinator species lists from the different sward types of the field experiment. Table S6. Species lists of pollinators found on each farm (2012 and 2013 surveys). Table S7. Correlations between the parameters of the pollinator communities in both the field experiment and farm pollinator surveys. [file JPE-53-906-s001.docx]

**Supplementary information**

**Grass only**

T

E†

T

P

T

MC

R

MC

T

E†

T

P

T

MC

R

MC

**Grass + legume**

T

MC

R

MC

T

MC

R

MC

**Grass + legume + forb**

T

P

T

MC

R

MC

T

P

T

MC

R

MC

**Graze**

**Cut**

Figure S1. Experimental design of one of the four replicate blocks from the DEFRA funded ‘Wide-scale Enhancement of Biodiversity Project’ from Rothamsted Research, North Wyke. T= Typical ELS managed plots. R= Rested plots. P=Ploughed. MC=Minimal cultivation. E†= Undisturbed existing grass sward. The highlighted plots represent those sampled from for this study encompassing the three different sward types (Grass, Grass-legumes, Grass-legume-forb) and two management types (cutting and grazing). The grazing management regime involved moderate grazing by cattle from April/May to early June, no grazing from early June to August to allow flowering, and moderate grazing by cattle from August to October (2 animals per plot (0.1 ha)). The cutting management regime involved one cut in early June and then moderate grazing by cattle from late August to October.

Prior to the experiment, the vegetation of the pasture corresponded to MG7 *Lolio-Cynosuretum* communities (Rodwell 1992). In the plots surveyed for the current study, minimum cultivation was used to create a surface tilth onto which the seed mixes were sown.

Surveying of the plots occurred over 48 days from 3 May-20 September in 2 week sampling rounds. Every two weeks each block was surveyed four times (each block was surveyed twice within a single day to account for insect day activity twice per sampling round). Over the entire field season each block was surveyed 24 times. Overall each seed mix was surveyed 192 times (4 replicate blocks and 2 plots of each sward type surveyed within each block). Each management type was surveyed 288 times (4 replicate blocks and 3 plots of each managment type surveyed within each block).

Figure S2. The entire layout of the field-experiment. The plots were spatially arranged to ensure that the treatments were not confounded by any edge effects. T= Typical ELS managed plots, R= Rested plots, P=Ploughed, MC=Minimal cultivation, E= Undisturbed existing grass sward, G= Grass (dark grey), GL= Grass-legumes (mid grey), GLF= Grass-legumes-forbs (white).


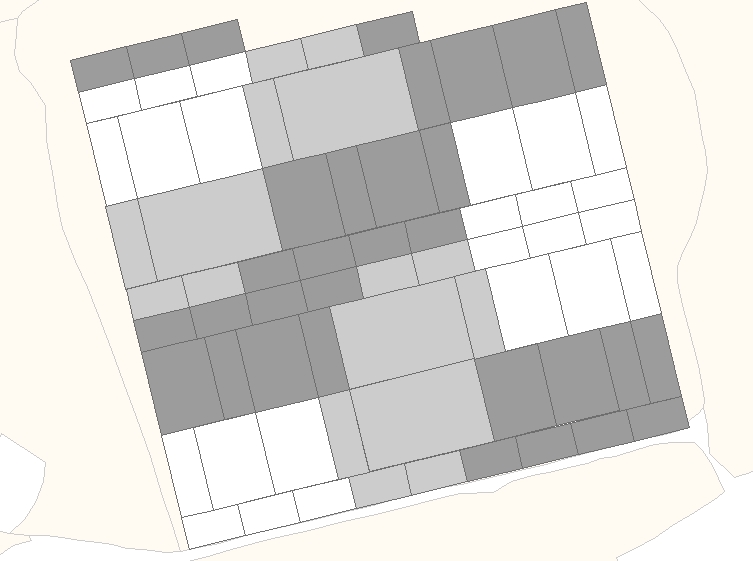


Block A

Block B

Block C

Block D

GLF

R MC

GLF

T P

GLF

T MC

GLF

T P

GLF

T MC

GLF

R MC

GLF

T MC

GLF

R MC

GLF

T P

GLF

T MC

GLF

T P

GLF

R MC

GLF

T P

GLF

T MC

GLF

R MC

GLF

R MC

GLF

T MC

GLF

T P

GLF

R MC

GLF

T P

GLF

T MC

GLF

R MC

GLF

T MC

GLF

T P

G

T MC

G

T E

G

T P

G

R MC

G

R MC

G

T P

G

T MC

G

T E

G

T P

G

T E

G

T MC

G

R MC

G

T P

G

T E

G

R MC

G

T MC

G

T E

G

T P

G

R MC

G

T MC

G

T E

G

T P

G

R MC

G

T MC

G

T P

G

T E

G

R MC

G

R MC

G

T MC

G

T E

G

T P

G

T MC

Graze

Graze

Cut

Cut

Graze

Graze

Cut

Cut

GL

R MC

GL

T MC

GL

R MC

GL

T MC

GL

T MC

GL

R MC

GL

R MC

GL

T MC

GL

T MC

GL

T MC

GL

R MC

GL

R MC

GL

R MC

GL

T MC

GL

R MC

GL

T MC

Block A

Block B

Block C

Block D

Graze

Graze

Cut

Cut

Graze

Graze

Cut

Cut

Cut

Figure S3. The locations of the 10 farms. The farms were all found in landscapes dominated by agriculture with some woodland. All were conventional mixed farms. The farms were used as they participated in a previous study (Macfadyen *et al.* 2009) so details of the farms were readily available. In Macfadyen and collagues’ study the farms were paired with organic farms and selected based on the fact that they were mixed farms with similar soil types and sizes.


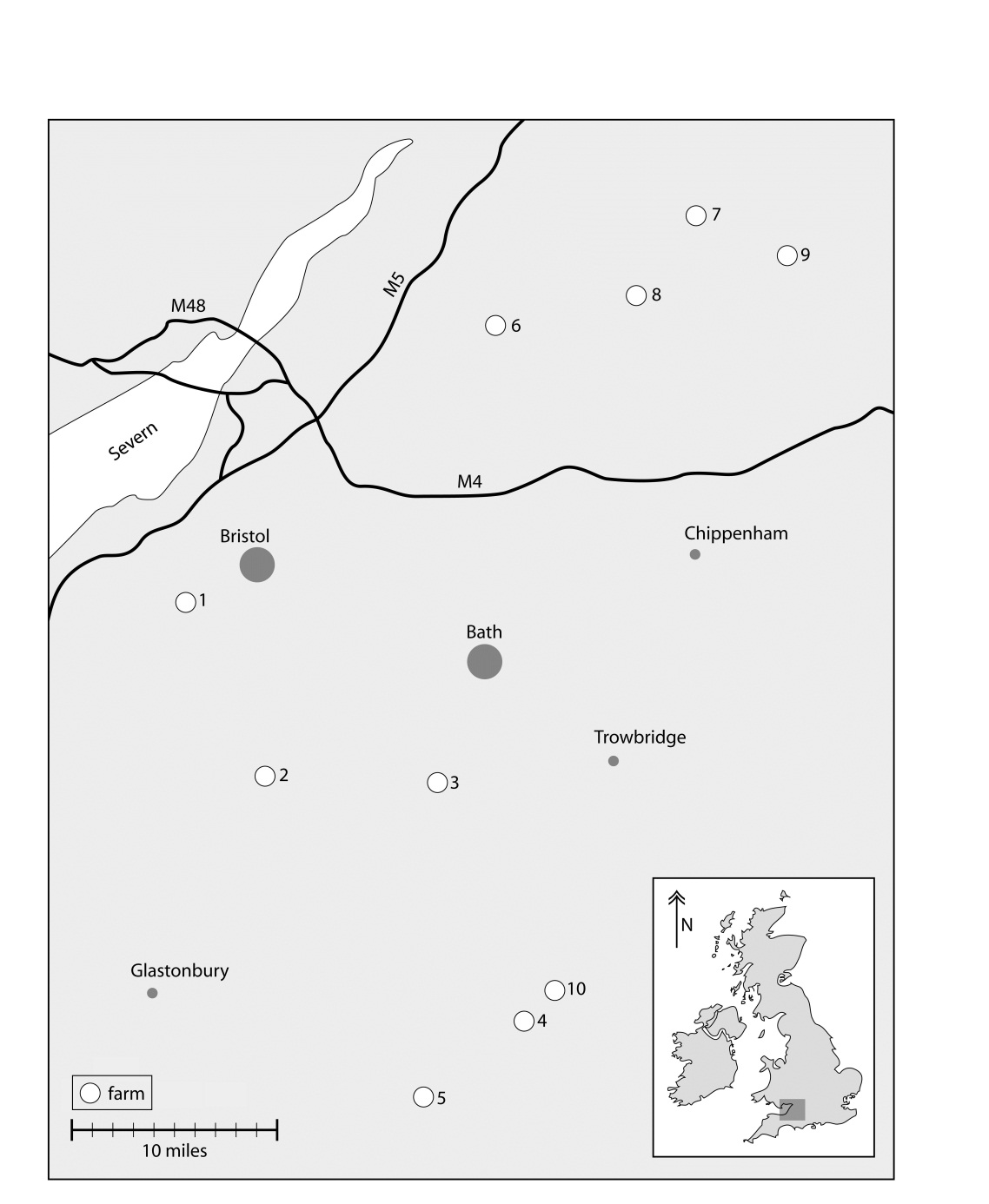


| **Farm** | **Grid reference** |
| --- | --- |
| 1 | ST52800, 69392 |
| 2 | ST58057, 54894 |
| 3 | ST71744, 55429 |
| 4 | ST77199, 36442 |
| 5 | ST69810, 30096 |
| 6 | ST76299, 90731 |
| 7 | ST92604, 99518 |
| 8 | ST87852, 93345 |
| 9 | ST99073, 98500 |
| 10 | ST79872, 38143 |

Figure S4. The grassland plant species’ interaction frequency and interaction richness (the number of pollinator visitors and the number of visitor species respectively) calculated from the plant-pollinator visitation network from a) the field experiment and b) the farm surveys. The number of interactions was divided by floral abundance. Species with an asterisk were those included in the seed mixes of the field experiment, all others established naturally.


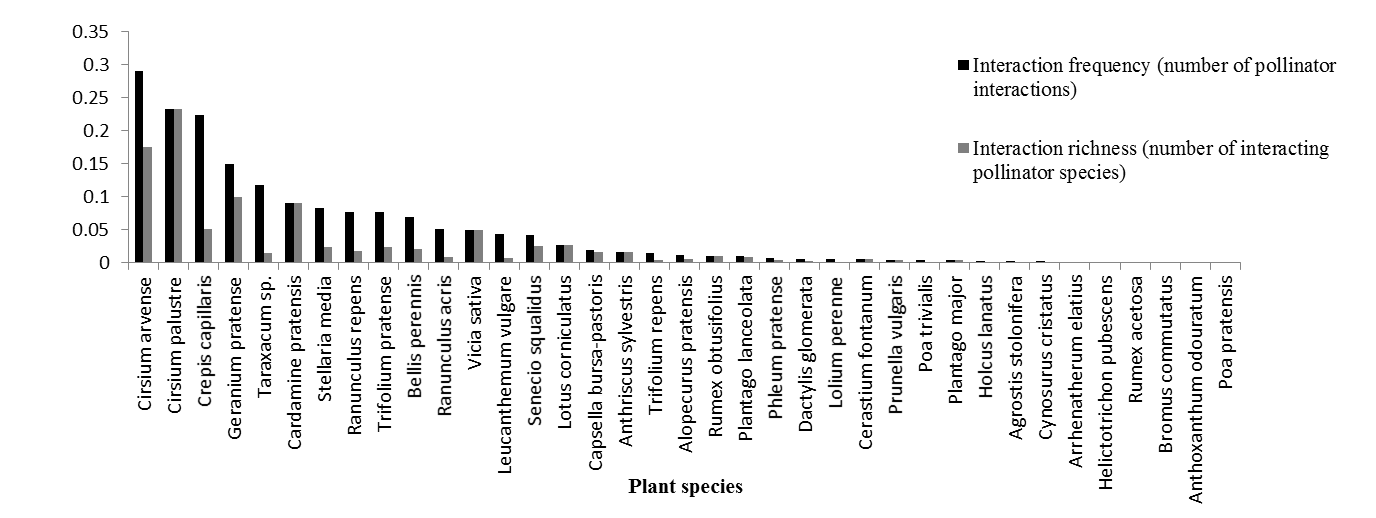


1)

2)


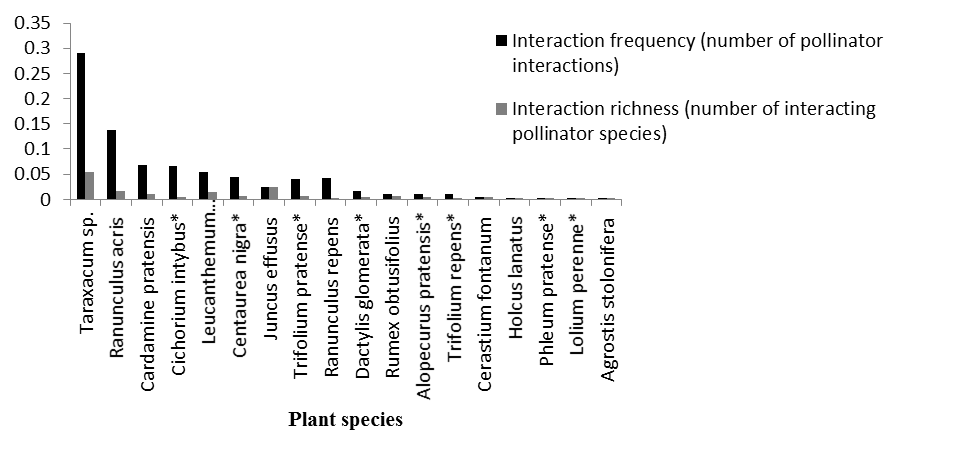


**Tables**

Table S1. The plant species of the three seed mixes sown in the plot experiment.

| **Grass only** | **Grass-legume** | **Grass-legume-forb** |
| --- | --- | --- |
| *Lolium perenne* (L.)  *Phleum pratense* (L.)  *Dactylis glomerata* (L.)  *Festuca pratensis* (Huds.)  *Alopecurus pratensis* (L*.*) | *Lolium perenne* (L.)  *Phleum pratense* (L.)  *Dactylis glomerata* (L.)  *Festuca pratensis* (Huds.)  *Alopecurus pratensis* (L*.*)  *Lotus corniculatus* (L*.*)  *Trifolium hybridum* (L*.*)  *Trifolium pratense* (L*.*)  *Trifolium repens* (L*.*)  *Onobrychis viciifolia* (Scop.)  *Melilotus officinalis* (L.)  *Medicago lupulina* (L.) | *Lolium perenne* (L.)  *Phleum pratense* (L.)  *Dactylis glomerata* (L.)  *Festuca pratensis* (Huds.)  *Alopecurus pratensis* (L*.*)  *Lotus corniculatus* (L*.*)  *Trifolium hybridum* (L*.*)  *Trifolium pratense* (L*.*)  *Trifolium repens* (L*.*)  *Onobrychis viciifolia* (Scop.)  *Melilotus officinalis* (L.)  *Medicago lupulina* (L.)  *Achillea millefolium* (L*.*)  *Centaurea nigra* (L.)  *Rumex acetosa* (L.)  *Sanguisorba minor* (Scop.)  *Cichorium intybus* (L.)  *Leucanthemum vulgare* (Lam.) |

Table S2. The realised composition of the three sward types under grazing or cutting management of the plot experiment (3 years following establishment of the seed mixes).

| **Grass only** | | **Grass-Legume** | | **Grass-Legume-Forb** | |
| --- | --- | --- | --- | --- | --- |
| **Grazed** | **Cut** | **Grazed** | **Cut** | **Grazed** | **Cut** |
| \| Agrostis stolonifera \| \| --- \| \| Alopecurus pratensis \| \| Cerastium fontanum \| \| Dactylis glomerata \| \| Holcus lanatus \| \| Lolium perenne \| \| Phleum pratense \| \| Ranunculus acris \| \| Ranunculus repens \| \| Rumex obtusifolius \| \| Trifolium repens \| | \| Agrostis stolonifera \| \| --- \| \| Cardamine pratensis \| \| Cerastium fontanum \| \| Dactylis glomerata \| \| Holcus lanatus \| \| Lolium perenne \| \| Phleum pratense \| \| Ranunculus acris \| \| Ranunculus repens \| \| Rumex obtusifolius \| \| Taraxacum off. \| \| Trifolium repens \| \| Veronica serpyllifolia \| | \| Agrostis stolonifera \| \| --- \| \| Cerastium fontanum \| \| Dactylis glomerata \| \| Holcus lanatus \| \| Lolium perenne \| \| Phleum pratense \| \| Ranunculus acris \| \| Ranunculus repens \| \| Rumex acetosa \| \| Rumex obtusifolius \| \| Taraxacum off. \| \| Trifolium pratense \| \| Trifolium repens \| \| Veronica serpyllifolia \| | \| Agrostis stolonifera \| \| --- \| \| Alopecurus pratensis \| \| Cerastium fontanum \| \| Dactylis glomerata \| \| Holcus lanatus \| \| Juncus effuses \| \| Lolium perenne \| \| Phleum pratense \| \| Ranunculus repens \| \| Rumex obtusifolius \| \| Taraxacum off. \| \| Trifolium pratense \| \| Trifolium repens \| \| Veronica serpyllifolia \| | \| Agrostis stolonifera \| \| --- \| \| Alopecurus pratensis \| \| Centaurea nigra \| \| Cerastium fontanum \| \| Cichorium intybus \| \| Cirsium vulgare \| \| Cynosaurus cristatus \| \| Dactylis glomerata \| \| Holcus lanatus \| \| Leucanthemum vulgare \| \| Lolium perenne \| \| Phleum pratense \| \| Ranunculus acris \| \| Ranunculus repens \| \| Rumex acetosa \| \| Rumex obtusifolius \| \| Sanguisorba minor \| \| Taraxacum off. \| \| Trifolium pratense \| \| Trifolium repens \| \| Veronica serpyllifolia \| | \| Agrostis stolonifera \| \| --- \| \| Alopecurus pratensis \| \| Bellis perennis \| \| Cardamine pratensis \| \| Centaurea nigra \| \| Cerastium fontanum \| \| Cichorium intybus \| \| Dactylis glomerata \| \| Holcus lanatus \| \| Leucanthemum vulgare \| \| Lolium perenne \| \| Lotus corniculatus \| \| Phleum pratense \| \| Ranunculus acris \| \| Ranunculus repens \| \| Rumex acetosa \| \| Rumex obtusifolius \| \| Taraxacum off. \| \| Trifolium pratense \| \| Trifolium repens \| |

Table S3. Plant species and their associated insect visitor species. The interactions were recorded during the surveys of the field-experiment and the farm surveys. When a pollinator was seen to visit a flower it was caught with a net and transferred to a killing tube. The pollinators were later pinned for identification by taxonomists and the following species lists created.

| **Agrostis stolonifera** | | | |
| --- | --- | --- | --- |
| **Plot experiment** | | **Field experiment** | |
| **Pollinator Species** | **Pollinator Order** | **Pollinator Species** | **Pollinator Order** |
| Bellardia vulgaris | Diptera | Melanostoma mellinum | Diptera |
| Botanophila striolata | Diptera | Anthomyia liturata | Diptera |
| Chloromyia formosa | Diptera | Scathophaga stercoraria | Diptera |
| Dilophus febrilis | Diptera | Rhagonycha fulva | Coleoptera |
| Episyrphus balteatus | Diptera | Sphaerophoria scripta | Diptera |
| Eupeodes corollae | Diptera | Rhagonycha fulva | Coleoptera |
| Hylemya vagans | Diptera |  |  |
| Ichneumonidae sp. | Hymenoptera |  |  |
| Melanostoma mellinum | Diptera |  |  |
| Pegoplata aestiva | Diptera |  |  |
| Platycheirus clypeatus | Diptera |  |  |
| Pollenia pediculata | Diptera |  |  |
| Pyralidae sp. | Lepidoptera |  |  |
| Rhagonycha fulva | Coleoptera |  |  |
| Scathophaga stercoraria | Diptera |  |  |
| Sphaerophoria interrupta | Diptera |  |  |

| **Alopecurus pratensis** | |
| --- | --- |
| **Field experiment** | |
| **Pollinator Species** | **Pollinator Order** |
| Botanophila striolata | Diptera |
| Chironomidae sp. | Diptera |
| Delia sp. | Diptera |
| Malachius bipustulatus | Coleoptera |
| Melanostoma mellinum | Diptera |
| Phyllobius pyri | Coleoptera |
| Scathophaga stercoraria | Diptera |

| **Anthoxanthum odouratum** | |
| --- | --- |
| **Field experiment** | |
| **Pollinator Species** | **Pollinator Order** |
| Rhagonycha fulva | Coleoptera |
| Melanostoma mellinum | Diptera |

| **Arrhenatherum elatius** | |
| --- | --- |
| **Field experiment** | |
| **Pollinator Species** | **Pollinator Order** |
| Opomyza germinationis | Diptera |
| Scathophaga stercoraria | Diptera |
| Platycheirus albimanus | Diptera |
| Rhagio tringarius | Diptera |

| **Anthriscus sylvestris** | |
| --- | --- |
| **Field experiment** | |
| **Pollinator Species** | **Pollinator Order** |
| Coccinella septempunctata | Coleoptera |

| **Bellis perennis** | |
| --- | --- |
| **Field experiment** | |
| **Pollinator Species** | **Pollinator Order** |
| Anthomyia liturata | Diptera |
| Botanophila striolata | Diptera |
| Delia platura | Diptera |
| Episyrphus balteatus | Diptera |
| Halictus tumulorum | Hymenoptera |
| Hydrellia griseola | Diptera |
| Hydrellia sp. | Diptera |
| Oscinella frit | Diptera |
| Pegoplata aestiva | Diptera |
| Platycheirus albimanus | Diptera |
| Rhamphomyia longipes | Diptera |
| Scathophaga stercoraria | Diptera |
| Siphona geniculata | Diptera |

| **Bromus commutatus** | |
| --- | --- |
| **Field experiment** | |
| **Pollinator Species** | **Pollinator Order** |
| Cantharis rustica | Coleoptera |
| Empis caudatula | Diptera |
| Empis punctata | Diptera |
| Tipula paludosa | Diptera |

| **Capsella bursa-pastoris** | |
| --- | --- |
| **Field experiment** | |
| **Pollinator Species** | **Pollinator Order** |
| Ceutorhynchus erysimi | Coleoptera |
| Lonchoptera bifurcata | Diptera |
| Scathophaga stercoraria | Diptera |
| Siphona geniculata | Diptera |

| **Cardamine pratensis** | | | |
| --- | --- | --- | --- |
| **Plot experiment** | | **Field experiment** | |
| **Pollinator species** | **Pollinator order** | **Pollinator species** | **Pollinator order** |
| Empis bicuspidata | Diptera | Botanophila striolata | Diptera |
|  |  | Adia cinerella | Diptera |
|  |  | Lasioglossum calceatum | Hymenoptera |

| **Centaurea nigra** | | |
| --- | --- | --- |
| **Plot experiment** | | |
| **Pollinator species** | **Pollinator order** | |
| Adia cinerella | Diptera | |
| Anthomyia liturata | Diptera | |
| Apis mellifera | Hymenoptera | |
| Bellardia vulgaris | Diptera | |
| Bombus lapidarius | Hymenoptera | |
| Bombus lucorum | Hymenoptera | |
| Bombus pascuorum | Hymenoptera | |
| Bombus pratorum | Hymenoptera | |
| Cetema paramyopina | Diptera | |
| Chloromyia formosa | Diptera | |
| Clostertomus norwegicus | Hemoptera | |
| Coenosia tigrina | Diptera | |
| Episyrphus balteatus | Diptera | |
| Eristalis abusivus | Diptera | |
| Eristalis arbustorum | Diptera | |
| Eristalis tenax | Diptera | |
| Geomyza tripunctata | Diptera | |
| Helophilus hybridus | Diptera | |
| Maniola jurtina | Lepidoptera | |
| Melanostoma mellinum | Diptera | |
| Meligethes sp. | Coleoptera | |
| Neomyia viridescens | Diptera | |
| Pegoplata aestiva | Diptera | |
| Platycheirus albimanus | Diptera | |
| Pollenia rudis | Diptera |  |
| Pyronia tithonus | Lepidoptera |  |
| Rhagonycha fulva | Coleoptera |  |
| Rhingia campestris | Diptera |  |
| Saltella sphondylii | Diptera |  |
| Scathophaga stercoraria | Diptera |  |
| Siphona urbana | Diptera |  |
| Sphaerophoria interrupta | Diptera |  |
| Syrphus ribesii | Diptera |  |

| **Cerastium fontanum** | | | |
| --- | --- | --- | --- |
| **Plot experiment** | | **Field experiment** | |
| **Pollinator Species** | **Pollinator Order** | **Pollinator Species** | **Pollinator Order** |
| Scathophaga stercoraria | Diptera | Botanophila striolata | Diptera |
|  |  | Braconidae sp. | Hymenoptera |
|  |  | Episyrphus balteatus | Diptera |
|  |  | Platycheirus albimanus | Diptera |
|  |  | Siphona geniculate | Diptera |

| **Cichorium intybus** | |
| --- | --- |
| **Plot experiment** | |
| **Pollinator Species** | **Pollinator Order** |
| Adia cinerella | Diptera |
| Apis mellifera | Hymenoptera |
| Azelia zetterstedti | Diptera |
| Bellardia vulgaris | Diptera |
| Bombus lapidarius | Hymenoptera |
| Bombus pascuorum | Hymenoptera |
| Bombus pratorum | Hymenoptera |
| Botanophila striolata | Diptera |
| Coenosia tigrina | Diptera |
| Dilophus febrilis | Diptera |
| Dolichopus plumipes | Diptera |
| Empis bicuspidata | Diptera |
| Episyrphus balteatus | Diptera |
| Eristalis arbustorum | Diptera |
| Eristalis tenax | Diptera |
| Eupeodes corollae | Diptera |
| Haematopota pluvialis | Diptera |
| Helophilus pendulus | Diptera |
| Lonchoptera furcata | Diptera |
| Maniola jurtina | Lepidoptera |
| Melanostoma mellinum | Diptera |
| Melanostoma scalare | Diptera |
| Meligethes sp. | Coleoptera |
| Muscina assimilis | Diptera |
| Oedemera lurida | Coleoptera |
| Opomyza germinationis | Diptera |
| Pegoplata aestiva | Diptera |
| Platycheirus albimanus | Diptera |
| Platycheirus clypeatus | Diptera |
| Platycheirus manicatus | Diptera |
| Platycheirus peltatus | Diptera |
| Pollenia rudis | Diptera |
| Rhingia campestris | Diptera |
| Scaeva pyrastri | Diptera |
| Scathophaga stercoraria | Diptera |
| Siphona urbana | Diptera |
| Sphaerophoria scripta | Diptera |
| Syrphus ribesii | Diptera |

| **Cirsium arvense** | |
| --- | --- |
| **Field experiment** | |
| **Pollinator Species** | **Pollinator Order** |
| Andrena dorsata | Hymenoptera |
| Bombus terrestris | Hymenoptera |
| Botanophila striolata | Diptera |
| Eriothrix rufomaculata | Diptera |
| Eristalinus sepulchralis | Diptera |
| Eristalis arbustorum | Diptera |
| Eristalis interruptus | Diptera |
| Eristalis tenax | Diptera |
| Helophilus trivittatus | Diptera |
| Melanostoma mellinum | Diptera |
| Meligethes sp. | Coleoptera |
| Neomyia cornicina | Diptera |
| Neomyia viridescens | Diptera |
| Rhagonycha fulva | Coleoptera |
| Saltella sphondylii | Diptera |
| Scathophaga stercoraria | Diptera |
| Syritta pipiens | Diptera |

| **Cirsium palustre** | |
| --- | --- |
| **Field experiment** | |
| **Pollinator Species** | **Pollinator Order** |
| Bombus pascuorum | Hymenoptera |
| Eristalis tenax | Diptera |
| Hylemya vagans | Diptera |
| Melanostoma scalare | Diptera |
| Platycheirus albimanus | Diptera |
| Scathophaga stercoraria | Diptera |
| **Cirsium vulgare** | |
| **Field experiment** | |
| **Pollinator Species** | **Pollinator Order** |
| Bombus lapidarius | Hymenoptera |
| Eristalis tenax | Diptera |
| Melanargia galathea | Lepidoptera |
| Scathophaga stercoraria | Diptera |

| **Crepis capillaris** | |
| --- | --- |
| **Field experiment** | |
| **Pollinator Species** | **Pollinator Order** |
| Apis mellifera | Hymenoptera |
| Chalcodoidea sp. | Hymenoptera |
| Empis femorata | Diptera |
| Meligethes sp. | Coleoptera |
| Odermera nobilis | Coleoptera |
| Oedemera lurida | Coleoptera |
| Pegoplata aestiva | Diptera |
| Pegoplata debilis | Diptera |
| Platycheirus albimanus | Diptera |
| Rhagonycha fulva | Coleoptera |

| **Cynosurus cristatus** | |
| --- | --- |
| **Field experiment** | |
| **Pollinator Species** | **Pollinator Order** |
| Delia sp. | Diptera |

| **Dactylis glomerata** | | | |
| --- | --- | --- | --- |
| **Plot experiment** | | **Field experiment** | |
| **Pollinator Species** | **Pollinator Order** | **Pollinator Species** | **Pollinator Order** |
| Bellardia pandia | Diptera | Athous bicolor | Coleoptera |
| Bellardia vulgaris | Diptera | Botanophila striolata | Diptera |
| Bombus terrestris | Hymenoptera | Coccinella septempunctata | Coleoptera |
| Botanophila striolata | Diptera | Delia platura | Diptera |
| Coccidula rufa | Coleoptera | Dilophus febrilis | Diptera |
| Coenosia tigrina | Diptera | Harmonia axyridis | Coleoptera |
| Dilophus febrilis | Diptera | Hylemya vagans | Diptera |
| Eupeodes sp. | Diptera | Ichneumon sp. | Hymenoptera |
| Helina duplicata | Diptera | Lotophila atra | Diptera |
| Hylemya variata | Diptera | Melanostoma mellinum | Diptera |
| Melanostoma mellinum | Diptera | Odermera nobilis | Coleoptera |
| Pegoplata aestiva | Diptera | Phyllobius pyri | Coleoptera |
| Platycheirus clypeatus | Diptera | Propylea quatuordecimpunctata | Coleoptera |
| Pollenia pediculata | Diptera | Rhagonycha fulva | Coleoptera |
| Pollenia rudis | Diptera | Scathophaga stercoraria | Diptera |
| Rhagonycha fulva | Coleoptera |  |  |
| Sarcophaga carnaria | Diptera |  |  |
| Scathophaga stercoraria | Diptera |  |  |

| **Festuca pratensis** | |
| --- | --- |
| **Plot experiment** | |
| **Pollinator Species** | **Pollinator Order** |
| Melanostoma mellinum | Diptera |

| **Geranium pratense** | |
| --- | --- |
| **Field experiment** | |
| **Pollinator Species** | **Pollinator Order** |
| Adia cinerella | Diptera |
| Pegoplata aestiva | Diptera |
|  | |
| **Helictotrichon pubescens** | |
| **Field experiment** | |
| **Pollinator Species** | **Pollinator Order** |
| Melanostoma mellinum | Diptera |
| Rhagonycha fulva | Coleoptera |

| **Heracleum sphondylium** | |
| --- | --- |
| **Field experiment** | |
| **Pollinator Species** | **Pollinator Order** |
| Cantharis rustica | Coleoptera |
| Ichneumon sp. | Hymenoptera |
| Pollenia pediculata | Diptera |

| **Holcus lanatus** | | | |
| --- | --- | --- | --- |
| **Plot experiment** | | **Field experiment** | |
| **Pollinator Species** | **Pollinator Order** | **Pollinator Species** | **Pollinator Order** |
| Anthomyia liturata | Diptera | Brontaea humilis | Diptera |
| Bellardia vulgaris | Diptera | Chaetorellia jaceae | Diptera |
| Botanophila striolata | Diptera | Coenosia tigrina | Diptera |
| Braconidae sp. | Hymenoptera | Delia platura | Diptera |
| Chloromyia formosa | Diptera | Dolichopus agilis | Diptera |
| Coenosia tigrina | Diptera | Episyrphus balteatus | Diptera |
| Delia sp. | Diptera | Fannia fuscula | Diptera |
| Dilophus febrilis | Diptera | Harmonia axyridis | Coleoptera |
| Dolichopus simplex | Diptera | Helina parcepilosa | Diptera |
| Helina communis | Diptera | Melanostoma mellinum | Diptera |
| Helina duplicata | Diptera | Meligethes sp. | Coleoptera |
| Hylemya vagans | Diptera | Melinda gentilis | Diptera |
| Melanostoma mellinum | Diptera | Oedemera nobilis | Coleoptera |
| Melanostoma scalare | Diptera | Pegoplata aestiva | Diptera |
| Muscina assimilis | Diptera | Pegoplata infirma | Diptera |
| Myospila meditabunda | Diptera | Rhagonycha fulva | Coleoptera |
| Neomyia cornicina | Diptera | Sarcophaga sp. | Diptera |
| Pegoplata aestiva | Diptera | Scathophaga stercoraria | Diptera |
| Platycheirus clypeatus | Diptera | Sciara hemerobioides | Diptera |
| Pollenia pediculata | Diptera |  |  |
| Pollenia rudis | Diptera |  |  |
| Ravinia pernix | Diptera |  |  |
| Rhagonycha fulva | Coleoptera |  |  |
| Sarcophaga sensu lato | Diptera |  |  |
| Scathophaga stercoraria | Diptera |  |  |
| Sphaerophoria sp. | Diptera |  |  |
| Tenthredo arcuata | Hymenoptera |  |  |

| **Juncus effusus** | |
| --- | --- |
| **Plot experiment** | |
| **Pollinator Species** | **Pollinator Order** |
| Pyralidae sp. | Lepidoptera |

| **Leucanthemum vulgare** | | | |
| --- | --- | --- | --- |
| **Plot experiment** | | **Field experiment** | |
| **Pollinator Species** | **Pollinator Order** | **Pollinator Species** | **Pollinator Order** |
| Adia cinerella | Diptera | Scathophaga stercoraria | Diptera |
| Anthomyia liturata | Diptera |  |  |
| Bellardia vulgaris | Diptera |  |  |
| Botanophila striolata | Diptera |  |  |
| Cheilosia vernalis | Diptera |  |  |
| Chrysotus sp. | Diptera |  |  |
| Coccinella septempunctata | Coleoptera |  |  |
| Coenosia tigrina | Diptera |  |  |
| Delia platura | Diptera |  |  |
| Empis albinervis | Diptera |  |  |
| Empis bicuspidata | Diptera |  |  |
| Episyrphus balteatus | Diptera |  |  |
| Eriothrix rufomaculata | Diptera |  |  |
| Eristalis arbustorum | Diptera |  |  |
| Eristalis tenax | Diptera |  |  |
| Eupeodes corollae | Diptera |  |  |
| Haematopota pluvialis | Diptera |  |  |
| Hylemya variata | Diptera |  |  |
| Melanostoma mellinum | Diptera |  |  |
| Meligethes sp. | Coleoptera |  |  |
| Neomyia viridescens | Diptera |  |  |
| Pegoplata aestiva | Diptera |  |  |
| Pollenia pediculata | Diptera |  |  |
| Pollenia rudis | Diptera |  |  |
| Pyralidae sp. | Lepidoptera |  |  |
| Rhagonycha fulva | Coleoptera |  |  |
| Saltella sphondylii | Diptera |  |  |
| Scathophaga stercoraria | Diptera |  |  |
| Siphona urbana | Diptera |  |  |
| Sphaerophoria interrupta | Diptera |  |  |
| Syritta pipiens | Diptera |  |  |
| Tenthredo arcuata | Hymenoptera |  |  |
| Urophora jaceana | Diptera |  |  |

| **Lolium perenne** | | | |
| --- | --- | --- | --- |
| **Plot experiment** | | **Field experiment** | |
| **Pollinator Species** | **Pollinator Order** | **Pollinator Species** | **Pollinator Order** |
| Acanthiophilus helianthi | Diptera | Amara similata | Coleoptera |
| Anthomyia liturata | Diptera | Anthomyia liturata | Diptera |
| Apis mellifera | Hymenoptera | Athous bicolor | Coleoptera |
| Bellardia pandia | Diptera | Botanophila fugax | Diptera |
| Bellardia vulgaris | Diptera | Chloromyia Formosa | Diptera |
| Botanophila striolata | Diptera | Fannia serena | Diptera |
| Calliphora vicina | Diptera | Hylemya vagans | Diptera |
| Chloromyia formosa | Diptera | Hylemya variata | Diptera |
| Coccinella septempunctata | Coleoptera | Lotophila atra | Diptera |
| Coenosia tigrina | Diptera | Melanostoma mellinum | Diptera |
| Delia platura | Diptera | Melanostoma scalare | Diptera |
| Dilophus febrilis | Diptera | Meligethes sp. | Coleoptera |
| Episyrphus balteatus | Diptera | Mydaea anicula | Diptera |
| Eristalis arbustorum | Diptera | Myospila meditabunda | Diptera |
| Eupeodes corollae | Diptera | Platycheirus albimanus | Diptera |
| Haematopota pluvialis | Diptera | Rhagonycha fulva | Coleoptera |
| Helina communis | Diptera | Scathophaga stercoraria | Diptera |
| Helina duplicata | Diptera | Sciara hemerobioides | Diptera |
| Helina obscurata | Diptera |  |  |
| Helina parcepilosa | Diptera |  |  |
| Hydrotaea cinerea | Diptera |  |  |
| Hylemya vagans | Diptera |  |  |
| Hylemya variata | Diptera |  |  |
| Melanostoma mellinum | Diptera |  |  |
| Melanostoma scalare | Diptera |  |  |
| Musca autumnalis | Diptera |  |  |
| Muscina assimilis | Diptera |  |  |
| Myospila meditabunda | Diptera |  |  |
| Neocrepidodera transversa | Coleoptera |  |  |
| Neomyia cornicina | Diptera |  |  |
| Pegoplata aestiva | Diptera |  |  |
| Philaenus spumarius | Hemoptera |  |  |
| Platycheirus clypeatus | Diptera |  |  |
| Platycheirus granditarsus | Diptera |  |  |
| Pollenia amentaria | Diptera |  |  |
| Pollenia pediculata | Diptera |  |  |
| Pollenia rudis | Diptera |  |  |
| Protapion trifolii | Coleoptera |  |  |
| Pyralidae sp. | Lepidoptera |  |  |
| Rhagonycha fulva | Coleoptera |  |  |
| Sarcophaga carnaria | Diptera |  |  |
| Sarcophaga melanura | Diptera |  |  |
| Sarcophaga sensu lato | Diptera |  |  |
| Sarcophaga sinuata | Diptera |  |  |
| Sarcophaga subvicina | Diptera |  |  |
| Sarcophaga variegata | Diptera |  |  |
| Scaeva pyrastri | Diptera |  |  |
| Scathophaga stercoraria | Diptera |  |  |
| Tenthredo arcuata | Hymenoptera |  |  |
| Tipula paludosa | Diptera |  |  |

| **Lotus corniculatus** | |
| --- | --- |
| **Field experiment** | |
| **Pollinator Species** | **Pollinator Order** |
| Eristalis arbustorum | Diptera |
| Geomyza majuscula | Diptera |
| Lasius niger | Hymenoptera |
| Meligethes sp. | Coleoptera |
| Oscinella frit | Diptera |
| Pegoplata aestiva | Diptera |
| Sphaerophoria scripta | Diptera |

| **Matricaria discoidea** | |
| --- | --- |
| **Field experiment** | |
| **Pollinator Species** | **Pollinator Order** |
| Hylemya variata | Diptera |
| Scathophaga stercoraria | Diptera |

| **Phleum pratense** | |
| --- | --- |
| **Field experiment** | |
| **Pollinator Species** | **Pollinator Order** |
| Helina setiventris | Diptera |
| Melanostoma mellinum | Diptera |
| Rhagonycha fulva | Coleoptera |

| **Plantago major** | |
| --- | --- |
| **Field experiment** | |
| **Pollinator Species** | **Pollinator Order** |
| Melanostoma mellinum | Diptera |

| **Poa pratense** | |
| --- | --- |
| **Field experiment** | |
| **Pollinator Species** | **Pollinator Order** |
| Scathophaga stercoraria | Diptera |

| **Poa trivialis** | |
| --- | --- |
| **Field experiment** | |
| **Pollinator Species** | **Pollinator Order** |
| Chloromyia formosa | Diptera |
| Geomyza tripunctata | Diptera |
| Hydrellia griseola | Diptera |
| Lonchoptera bifurcata | Diptera |
| Lonchoptera lutea | Diptera |
| Lotophila atra | Diptera |
| Phytomyza notata | Diptera |
| Scathophaga stercoraria | Diptera |

| **Prunella vulgaris** | |
| --- | --- |
| **Field experiment** | |
| **Pollinator Species** | **Pollinator Order** |
| Apis melifera | Hymenoptera |

| **Ranunculus acris** | | | |
| --- | --- | --- | --- |
| **Plot experiment** | | **Field experiment** | |
| **Pollinator Species** | **Pollinator Order** | **Pollinator Species** | **Pollinator Order** |
| Anthomyia liturata | Diptera | Apis mellifera | Hymenoptera |
| Botanophila striolata | Diptera | Adia cinerella | Diptera |
| Cetema paramyopina | Diptera | Botanophila striolata | Diptera |
| Chloromyia formosa | Diptera | Braconidae sp. | Hymenoptera |
| Chrysotus sp. | Diptera | Cantharis rustica | Coleoptera |
| Delia sp. | Diptera | Cheilosia albitarsis | Diptera |
| Empis bicuspidata | Diptera | Cheilosia latifrons | Diptera |
| Episyrphus balteatus | Diptera | Copromyza similis | Diptera |
| Hylemya vagans | Diptera | Delia sp. | Diptera |
| Melanostoma mellinum | Diptera | Dioctria atricapilla | Diptera |
| Meligethes sp. | Coleoptera | Empis albinervis | Diptera |
| Meliscaeva auricollis | Diptera | Empis caudatula | Diptera |
| Pegoplata aestiva | Diptera | Episyrphus balteatus | Diptera |
| Pegoplata infirma | Diptera | Halictus tumulorum | Hymenoptera |
| Saltella sphondylii | Diptera | Harmonia axyridis | Coleoptera |
| Scathophaga stercoraria | Diptera | Hylemya vagans | Diptera |
| Siphona urbana | Diptera | Hylemya variata | Diptera |
| Sphaerophoria interrupta | Diptera | Lasioglossum calceatum | Hymenoptera |
| Tenthredo arcuata | Hymenoptera | Lotophila atra | Diptera |
|  |  | Melanostoma mellinum | Diptera |
|  |  | Meligethes sp. | Coleoptera |
|  |  | Merodon equestris | Hymenoptera |
|  |  | Myrmica ruginodis | Hymenoptera |
|  |  | Oedemera nobilis | Coleoptera |
|  |  | Oscinella frit | Larvae |
|  |  | Pegoplata aestival | Diptera |
|  |  | Pegoplata debilis | Diptera |
|  |  | Pegoplata infirma | Diptera |
|  |  | Platycheirus albimanus | Diptera |
|  |  | Protapion abricans | Coleoptera |
|  |  | Protapion trifolii | Coleoptera |
|  |  | Rhagio scolopaceus | Diptera |
|  |  | Rhagio tringarius | Diptera |
|  |  | Rhamphomyia longipes | Diptera |
|  |  | Scathophaga stercoraria | Diptera |
|  |  | Sepsis fulgens | Diptera |
|  |  | Sphaerophoria scripta | Diptera |
|  |  | Syritta pipiens | Diptera |

| **Ranunculus repens** | | | |
| --- | --- | --- | --- |
| **Plot experiment** | | **Field experiment** | |
| **Pollinator Species** | **Pollinator Order** | **Pollinator Species** | **Pollinator Order** |
| Adia cinerella | Diptera | Andrena nitida | Hymenoptera |
| Andrena nigroaenea | Hymenoptera | Andrena cineraria | Hymenoptera |
| Anthomyia liturata | Diptera | Botanophila fugax | Diptera |
| Apis mellifera | Hymenoptera | Botanophila striolata | Diptera |
| Bellardia vulgaris | Diptera | Braconidae sp. | Hymenoptera |
| Bicellaria sulcata | Diptera | Cheilosia albitarsis | Diptera |
| Bombus lapidarius | Hymenoptera | Chironomidae sp. | Diptera |
| Bombus lucorum | Hymenoptera | Copromyza similis | Diptera |
| Bombus pratorum | Hymenoptera | Delia platura | Diptera |
| Botanophila striolata | Diptera | Dolichopus plumipes | Diptera |
| Cantharis nigra | Coleoptera | Empidiae sp. | Diptera |
| Cetema neglecta | Diptera | Empis albinervis | Diptera |
| Cetema paramyopina | Diptera | Empis livida | Diptera |
| Cheilosia albitarsis | Diptera | Empis tumida | Diptera |
| Cheilosia latifrons | Diptera | Episyrphus balteatus | Diptera |
| Cheilosia vernalis | Diptera | Eristalis arbustorum | Diptera |
| Chloromyia formosa | Diptera | Eudasyphora cyanicolor | Diptera |
| Chromatomyia nigra | Diptera | Lasioglossum calceatum | Hymenoptera |
| Chrysotus sp. | Diptera | Melanostoma mellinum | Diptera |
| Coccidula rufa | Coleoptera | Melanostoma scalare | Diptera |
| Coccinella septempunctata | Coleoptera | Meligethes sp. | Coleoptera |
| Coenosia tigrina | Diptera | Merodon equestris | Hymenoptera |
| Coproica ferruginea | Diptera | Oedemera nobilis | Coleoptera |
| Copromyza similis | Diptera | Pegoplata aestival | Diptera |
| Delia florilega | Diptera | Pegoplata infirma | Diptera |
| Delia platura | Diptera | Pherbellia cinerella | Diptera |
| Dilophus febrilis | Diptera | Platycheirus albimanus | Diptera |
| Diplonevra funebris | Diptera | Rhamphomyia longipes | Diptera |
| Diplonevra sp. | Diptera | Saltella sphondylii | Diptera |
| Dolichopus caligatus | Diptera | Scathophaga stercoraria | Diptera |
| Dolichopus plumipes | Diptera | Siphona geniculate | Diptera |
| Empis albinervis | Diptera |  |  |
| Empis bicuspidata | Diptera |  |  |
| Empis livida | Diptera |  |  |
| Episyrphus balteatus | Diptera |  |  |
| Eriothrix rufomaculata | Diptera |  |  |
| Eristalis arbustorum | Diptera |  |  |
| Eupeodes corollae | Diptera |  |  |
| Eupeodes latifasciatus | Diptera |  |  |
| Fannia serena | Diptera |  |  |
| Geomyza tripunctata | Diptera |  |  |
| Glyphipterix simpliciella | Lepidoptera |  |  |
| Haematopota pluvialis | Diptera |  |  |
| Helina communis | Diptera |  |  |
| Helina parcepilosa | Diptera |  |  |
| Hydrellia sp. | Diptera |  |  |
| Hylemya vagans | Diptera |  |  |
| Hylemya variata | Diptera |  |  |
| Lejogaster metallina | Diptera |  |  |
| Lonchoptera furcata | Diptera |  |  |
| Lonchoptera lutea | Diptera |  |  |
| Lotophila atra | Diptera |  |  |
| Malachius bipustulatus | Coleoptera |  |  |
| Melanostoma mellinum | Diptera |  |  |
| Melanostoma scalare | Diptera |  |  |
| Meligethes sp. | Coleoptera |  |  |
| Metopina sp. | Diptera |  |  |
| Micropterix calthella | Lepidoptera |  |  |
| Myospila meditabunda | Diptera |  |  |
| Oedemera nobilis | Coleoptera |  |  |
| Opomyza germinationis | Diptera |  |  |
| Oscinella frit | Diptera |  |  |
| Oscinella nitidissima | Diptera |  |  |
| Pegoplata aestiva | Diptera |  |  |
| Phaonia incana | Diptera |  |  |
| Phytomyza vitalbae | Diptera |  |  |
| Platycheirus albimanus | Diptera |  |  |
| Platycheirus clypeatus | Diptera |  |  |
| Platycheirus europaeus | Diptera |  |  |
| Pollenia labialis | Diptera |  |  |
| Pollenia rudis | Diptera |  |  |
| Propylea quatuordecimpunctata | Coleoptera |  |  |
| Protapion fulvipes | Coleoptera |  |  |
| Rhagio scolapaceus | Diptera |  |  |
| Rhagio tringarius | Diptera |  |  |
| Rhagonycha fulva | Coleoptera |  |  |
| Rhingia campestris | Diptera |  |  |
| Saltella sphondylii | Diptera |  |  |
| Scathophaga stercoraria | Diptera |  |  |
| Siphona urbana | Diptera |  |  |
| Spelobia sp. | Diptera |  |  |
| Sphaerophoria interrupta | Diptera |  |  |
| Sphaerophoria scripta | Diptera |  |  |
| Tachinus sp. | Coleoptera |  |  |
| Tenthredo arcuata | Hymenoptera |  |  |
|  |  |  |  |

| **Rumex acetosa** | |
| --- | --- |
| **Field experiment** | |
| **Pollinator Species** | **Pollinator Order** |
| Pollenia rudis | Diptera |

| **Rumex obtusifolius** | | | |
| --- | --- | --- | --- |
| **Plot experiment** | | **Field experiment** | |
| **Pollinator Species** | **Pollinator Order** | **Pollinator Species** | **Pollinator Order** |
| Agromyza sp. | Diptera | Melanostoma scalare | Diptera |
| Cantharis lateralis | Coleoptera |  |  |
| Cantharis nigra | Coleoptera |  |  |
| Cetema paramyopina | Diptera |  |  |
| Chloromyia formosa | Diptera |  |  |
| Coccinella septempunctata | Coleoptera |  |  |
| Gastrophysa viridula | Coleoptera |  |  |
| Geomyza tripunctata | Diptera |  |  |
| Haematopota pluvialis | Diptera |  |  |
| Pegoplata aestiva | Diptera |  |  |
| Platycheirus clypeatus | Diptera |  |  |
| Pollenia rudis | Diptera |  |  |
| Rhagonycha fulva | Coleoptera |  |  |
| Scathophaga stercoraria | Diptera |  |  |
|  |  |  |  |

| **Senecio squalidus** | |
| --- | --- |
| **Field experiment** | |
| **Pollinator Species** | **Pollinator Order** |
| Bombus terrestris | Hymenoptera |
| Odermera nobilis | Coleoptera |
| Rhagonycha fulva | Coleoptera |

| **Stellaria media** | |
| --- | --- |
| **Field experiment** | |
| **Pollinator Species** | **Pollinator Order** |
| Chalcidoidea sp. | Hymenoptera |
| Delia platura | Diptera |
| Lonchoptera bifurcata | Diptera |
| Meligethes sp. | Diptera |
| Microcercis albipalpis | Diptera |
| Oscinella frit | Diptera |
| Scathophaga stercoraria | Diptera |

| **Taraxacum sp.** | | | |
| --- | --- | --- | --- |
| **Plot experiment** | | **Field experiment** | |
| **Pollinator Species** | **Pollinator Order** | **Pollinator Species** | **Pollinator Order** |
| Anthomyia liturata | Diptera | Adia cinerella | Diptera |
| Bellardia vulgaris | Diptera | Agriotes sputator | Coleoptera |
| Bombus lapidarius | Hymenoptera | Andrena cineraria | Hymenoptera |
| Bombus pratorum | Hymenoptera | Aphthona euphorbiae | Coleoptera |
| Botanophila striolata | Diptera | Bombus lapidarius | Hymenoptera |
| Cantharis nigra | Coleoptera | Botanophila striolata | Diptera |
| Cetema paramyopina | Diptera | Braconidae sp. | Hymenoptera |
| Coccinella septempunctata | Coleoptera | Cecidomyidae sp. | Diptera |
| Diplonevra funebris | Diptera | Ceutorhynchus obstrictus | Coleoptera |
| Dolichopus caligatus | Diptera | Chalcodoidea sp. | Hymenoptera |
| Episyrphus balteatus | Diptera | Chironomidae sp. | Coleoptera |
| Eriothrix rufomaculata | Diptera | Copromyza equina | Diptera |
| Eupeodes corollae | Diptera | Delia platura | Diptera |
| Eupeodes latifasciatus | Diptera | Empis aemula | Diptera |
| Glyphipterix simpliciella | Lepidoptera | Empis caudatula | Diptera |
| Lasioglossum calceatum | Hymenoptera | Empis femorata | Diptera |
| Lonchoptera furcata | Diptera | Empis opaca | Diptera |
| Maniola jurtina | Lepidoptera | Episyrphus balteatus | Diptera |
| Megaselia sp. | Diptera | Eristalis arbustorum | Diptera |
| Melanostoma mellinum | Diptera | Eucoilidae sp. | Hymenoptera |
| Melanostoma scalare | Diptera | Fannia mollissima | Diptera |
| Meligethes sp. | Coleoptera | Hydrellia griseola | Diptera |
| Neocrepidodera transversa | Coleoptera | Hylemya variata | Diptera |
| Oedemera nobilis | Coleoptera | Lasioglossum puncticolle | Hymenoptera |
| Opomyza germinationis | Diptera | Lasius niger | Hymenoptera |
| Oscinella frit | Diptera | Lonchoptera bifurcata | Diptera |
| Oscinella nitidissima | Diptera | Lotophila atra | Diptera |
| Pegoplata aestiva | Diptera | Malachius bipustulatus | Coleoptera |
| Rhagio scolapaceus | Diptera | Megaselia sp. | Diptera |
| Saltella sphondylii | Diptera | Melanostoma mellinum | Diptera |
| Scathophaga stercoraria | Diptera | Melanostoma scalare | Diptera |
| Siphona urbana | Diptera | Meligethes sp. | Coleoptera |
| Sphaerophoria sp. interrupta | Diptera | Meoneura sp. | Diptera |
| Tenthredo arcuata | Hymenoptera | Neoascia podagrica | Diptera |
|  |  | Oscinella frit | Diptera |
|  |  | Oscinella nigerrima | Diptera |
|  |  | Pegoplata aestival | Diptera |
|  |  | Phyllobius pyri | Coleoptera |
|  |  | Platycheirus albimanus | Diptera |
|  |  | Pollenia amentaria | Diptera |
|  |  | Reichertella geniculata | Diptera |
|  |  | Rhamphomyia sulcata | Diptera |
|  |  | Saltella sphondylii | Diptera |
|  |  | Sarcophaga variegata | Diptera |
|  |  | Scathophaga stercoraria | Diptera |
|  |  | Sciara hemerobioides | Diptera |
|  |  | Sepsis cynipsea | Hymenoptera |
|  |  | Siphona geniculata | Diptera |
|  |  | Spelobia sp | Diptera |

| **Trifolium hybridium** | |
| --- | --- |
| **Plot experiment** | |
| **Pollinator Species** | **Pollinator Order** |
| Protapion fulvipes | Coleoptera |

| **Trifolium pratense** | | | |
| --- | --- | --- | --- |
| **Field experiment** | | **Plot experiment** | |
| **Pollinator Species** | **Pollinator Order** | **Pollinator Species** | **Pollinator Order** |
| Apis mellifera | Hymenoptera | Bellardia vulgaris | Diptera |
| Bombus hortorum | Hymenoptera | Bombus pascuorum | Hymenoptera |
| Bombus lapidarius | Hymenoptera | Bombus terrestris | Hymenoptera |
| Bombus lucorum | Hymenoptera | Chloromyia formosa | Diptera |
| Bombus pascuorum | Hymenoptera | Dilophus febrilis | Diptera |
| Bombus terrestris | Hymenoptera | Megaselia sp. | Diptera |
| Cortinicara gibbosa | Coleoptera | Melanostoma mellinum | Diptera |
| Halictus tumulorum | Hymenoptera | Meligethes sp. | Coleoptera |
| Hypera meles | Coleoptera | Pegoplata aestiva | Diptera |
| Lonchoptera bifurcata | Diptera | Platycheirus albimanus | Diptera |
| Meligethes sp. | Coleoptera | Protapion apricans | Coleoptera |
| Protapion abricans | Coleoptera | Protapion fulvipes | Coleoptera |
| Protapion fulvipes | Coleoptera | Protapion trifolii | Coleoptera |
| Protapion trifolii | Coleoptera | Rhingia campestris | Diptera |
| Scathophaga stercoraria | Diptera |  |  |

| **Trifolium repens** | | | |
| --- | --- | --- | --- |
| **Plot experiment** | | **Field experiment** | |
| **Pollinator Species** | **Pollinator Order** | **Pollinator Species** | **Pollinator Order** |
| Anthomyia liturata | Diptera | Andrena wilkella | Hymenoptera |
| Apis mellifera | Hymenoptera | Apis melifera | Hymenoptera |
| Bombus hortorum | Hymenoptera | Bombus lapidarius | Hymenoptera |
| Bombus lapidarius | Hymenoptera | Bombus lucorum | Hymenoptera |
| Bombus lucorum | Hymenoptera | Bombus pascuorum | Hymenoptera |
| Bombus pratorum | Hymenoptera | Bombus pratorum | Hymenoptera |
| Cetema neglecta | Diptera | Bombus terrestris | Hymenoptera |
| Empis livida | Diptera | Bombus vestalis | Hymenoptera |
| Episyrphus balteatus | Diptera | Botanophila striolata | Coleoptera |
| Eupeodes corollae | Diptera | Braconidae sp. | Hymenoptera |
| Helina obscurata | Diptera | Chironomidae sp. | Diptera |
| Ischnopterapion loti | Coleoptera | Empis livida | Diptera |
| Ischnopterapion virens | Coleoptera | Episyrphus balteatus | Diptera |
| Meligethes sp. | Coleoptera | Eupeodes luniger | Diptera |
| Neocrepidodera transversa | Coleoptera | Halictus tumulorum | Hymenoptera |
| Oedemera nobilis | Coleoptera | Harmonia axyridis | Coleoptera |
| Oscinella frit | Diptera | Melanostoma mellinum | Diptera |
| Pegoplata aestiva | Diptera | Meligethes sp. | Coleoptera |
| Protapion apricans | Coleoptera | Melitta leporine | Hymenoptera |
| Protapion fulvipes | Coleoptera | Pegoplata aestival | Coleoptera |
| Pyralidae sp. | Lepidoptera | Platycheirus albimanus | Diptera |
| Scathophaga stercoraria | Diptera | Protapion fulvipes | Coleoptera |
| Tipula paludosa | Diptera | Protapion trifolii | Coleoptera |
|  |  | Scathophaga stercoraria | Diptera |
|  |  | Sepsis sp. | Diptera |

| **Vicia sativa** | |
| --- | --- |
| **Field experiment** | |
| **Pollinator Species** | **Pollinator Order** |
| Cantharis nigra | Coleoptera |

Table S4. Field sizes of the two pasture fields per farm.

| **Farm** | **Field size (ha)** |
| --- | --- |
| 1 | 5.4 |
| 1 | 5.08 |
| 2 | 0.57 |
| 2 | 3.78 |
| 3 | 3.58 |
| 3 | 2.58 |
| 4 | 6.52 |
| 4 | 3.18 |
| 5 | 2.05 |
| 5 | 4.06 |
| 6 | 2.57 |
| 6 | 4.05 |
| 7 | 4.17 |
| 7 | 2.08 |
| 8 | 7.04 |
| 8 | 3.02 |
| 9 | 5.84 |
| 9 | 6.17 |
| 10 | 5.3 |
| 10 | 5.03 |

Table S5. Pollinator species lists from the plots of the field-experiment under the different seed mixes (grass only, grass-legume, grass-legume-forb) and management (cut/grazed).

|  | **Grass-legume-forb** | | **Grass-Legume** | | **Grass** | |
| --- | --- | --- | --- | --- | --- | --- |
| **Cut/**  **Grazed** | **Pollinator species** | **Pollinator order** | **Pollinator species** | **Pollinator order** | **Pollinator species** | **Pollinator order** |
| C | Adia cinerella | Diptera | Agromyza sp. | Diptera | Anthomyia liturata | Diptera |
| C | Anthomyia liturata | Diptera | Anthomyia liturata | Diptera | Apis mellifera | Hymenoptera |
| C | Apis mellifera | Hymenoptera | Apis mellifera | Hymenoptera | Bellardia vulgaris | Diptera |
| C | Bellardia vulgaris | Diptera | Bellardia vulgaris | Diptera | Bicellaria sulcata | Diptera |
| C | Bombus lapidarius | Hymenoptera | Bombus hortorum | Hymenoptera | Bombus lapidarius | Hymenoptera |
| C | Bombus lucorum | Hymenoptera | Bombus lapidarius | Hymenoptera | Botanophila striolata | Diptera |
| C | Bombus pascuorum | Hymenoptera | Bombus lucorum | Hymenoptera | Cetema neglecta | Diptera |
| C | Bombus pratorum | Hymenoptera | Bombus pascuorum | Hymenoptera | Cetema paramyopina | Diptera |
| C | Botanophila striolata | Diptera | Bombus terrestris | Hymenoptera | Cheilosia albitarsis | Diptera |
| C | Cetema neglecta | Diptera | Botanophila striolata | Diptera | Cheilosia latifrons | Diptera |
| C | Cheilosia albitarsis | Diptera | Cetema paramyopina | Diptera | Cheilosia vernalis | Diptera |
| C | Coenosia tigrina | Diptera | Cheilosia albitarsis | Diptera | Chloromyia formosa | Diptera |
| C | Delia platura | Diptera | Cheilosia griseiventris | Diptera | Chrysotus sp. | Diptera |
| C | Dilophus febrilis | Diptera | Chloromyia formosa | Diptera | Coccidula rufa | Coleoptera |
| C | Diplonevra funebris | Diptera | Coccinella septempunctata | Coleoptera | Coccinella septempunctata | Coleoptera |
| C | Dolichopus caligatus | Diptera | Coenosia tigrina | Diptera | Coenosia tigrina | Diptera |
| C | Dolichopus plumipes | Diptera | Dilophus febrilis | Diptera | Delia sp. | Diptera |
| C | Empis bicuspidata | Diptera | Diplonevra funebris | Diptera | Dilophus febrilis | Diptera |
| C | Episyrphus balteatus | Diptera | Dolichopus caligatus | Diptera | Dolichopus caligatus | Diptera |
| C | Eristalis abusivus | Diptera | Empis bicuspidata | Diptera | Dolichopus simplex | Diptera |
| C | Eristalis arbustorum | Diptera | Empis livida | Diptera | Empis bicuspidata | Diptera |
| C | Eristalis tenax | Diptera | Episyrphus balteatus | Diptera | Empis livida | Diptera |
| C | Eupeodes corollae | Diptera | Eupeodes corollae | Diptera | Episyrphus balteatus | Diptera |
| C | Eupeodes latifasciatus | Diptera | Eupeodes latifasciatus | Diptera | Eriothrix rufomaculata | Diptera |
| C | Fannia serena | Diptera | Geomyza tripunctata | Diptera | Glyphipterix simpliciella | Lepidoptera |
| C | Glyphipterix simpliciella | Lepidooptera | Glyphipterix simpliciella | Lepidoptera | Helina communis | Diptera |
| C | Haematopota pluvialis | Diptera | Haematopota pluvialis | Diptera | Helina duplicata | Diptera |
| C | Helina parcepilosa | Diptera | Helina duplicata | Diptera | Helina parcepilosa | Diptera |
| C | Hylemya vagans | Diptera | Helina obscurata | Diptera | Hylemya vagans | Diptera |
| C | Hylemya variata | Diptera | Hylemya vagans | Diptera | Hylemya variata | Diptera |
| C | Latridiidae sp. | Coleoptera | Ischnopterapion virens | Coleoptera | Lonchoptera furcata | Diptera |
| C | Malachius bipustulatus | Coleoptera | Lasioglossum calceatum | Hymenoptera | Lonchoptera lutea | Diptera |
| C | Megaselia sp. | Diptera | Lejogaster metallina | Diptera | Lucilia sericata | Diptera |
| C | Melanostoma mellinum | Diptera | Lonchoptera furcata | Diptera | Melanostoma mellinum | Diptera |
| C | Meligethes sp. | Coleoptera | Lonchoptera lutea | Diptera | Meligethes sp. | Coleoptera |
| C | Micropterix calthella | Lepidooptera | Maniola jurtina | Lepidoptera | Metopina sp. | Coleoptera |
| C | Muscina assimilis | Diptera | Megaselia sp. | Diptera | Micropterix calthella | Lepidoptera |
| C | Myospila meditabunda | Diptera | Melanostoma mellinum | Diptera | Muscina assimilis | Diptera |
| C | Oedemera nobilis | Coleoptera | Melanostoma scalare | Diptera | Neocrepidodera transversa | Coleoptera |
| C | Oscinella sp. | Diptera | Meligethes sp. | Coleoptera | Neophilaneus sp. | Coleoptera |
| C | Pegoplata aestiva | Diptera | Micropterix calthella | Lepidoptera | Oscinella nitidissima | Diptera |
| C | Pegoplata infirma | Diptera | Musca autumnalis | Diptera | Pegoplata aestiva | Diptera |
| C | Phaonia incana | Diptera | Muscina assimilis | Diptera | Phaonia incana | Diptera |
| C | Phytomyza vitalbae | Diptera | Neocrepidodera transversa | Coleoptera | Platycheirus clypeatus | Diptera |
| C | Platycheirus albimanus | Diptera | Oedemera nobilis | Coleoptera | Pollenia amentaria | Diptera |
| C | Platycheirus clypeatus | Diptera | Opomyza germinationis | Diptera | Pollenia pediculata | Diptera |
| C | Platycheirus peltatus | Diptera | Oscinella frit | Diptera | Pollenia rudis | Diptera |
| C | Pollenia pediculata | Diptera | Oscinella nitidissima | Diptera | Propylea quatuordecimpunctata | Coleoptera |
| C | Pollenia rudis | Diptera | Pegoplata aestiva | Diptera | Protapion fulvipes | Coleoptera |
| C | Protapion apricans | Coleoptera | Phaonia incana | Diptera | Protapion trifolii | Coleoptera |
| C | Protapion fulvipes | Coleoptera | Platycheirus albimanus | Diptera | Rhagonycha fulva | Diptera |
| C | Protapion trifolii | Coleoptera | Platycheirus angustatus | Diptera | Saltella sphondylii | Diptera |
| C | Rhagio scolapaceus | Diptera | Platycheirus clypeatus | Diptera | Sarcophaga carnaria | Diptera |
| C | Rhagonycha fulva | Coleoptera | Platycheirus sp. | Diptera | Sarcophaga sensu lato | Diptera |
| C | Rhingia campestris | Diptera | Pollenia pediculata | Diptera | Sarcophaga sinuata | Diptera |
| C | Saltella sphondylii | Diptera | Pollenia rudis | Diptera | Scaeva pyrastri | Diptera |
| C | Sarcophaga melanura | Diptera | Protapion apricans | Coleoptera | Scathophaga stercoraria | Diptera |
| C | Sarcophaga variegata | Diptera | Protapion fulvipes | Coleoptera | Siphona urbana | Diptera |
| C | Scaeva pyrastri | Diptera | Protapion trifolii | Coleoptera | Sphaerophoria interrupta | Diptera |
| C | Scathophaga stercoraria | Diptera | Pyralidae sp. | Lepidoptera | Tenthredo arcuata | Hymenoptera |
| C | Siphona urbana | Diptera | Ravinia pernix | Diptera |  |  |
| C | Sphaerophoria interrupta | Diptera | Rhagio scolapaceus | Diptera |  |  |
| C | Sphaerophoria scripta | Diptera | Rhagio tringarius | Diptera |  |  |
| C | Tenthredo arcuata | Hymenoptera | Rhagonycha fulva | Diptera |  |  |
| C | Tipula paludosa | Diptera | Rhingia campestris | Diptera |  |  |
| C |  |  | Saltella sphondylii | Diptera |  |  |
| C |  |  | Sarcophaga carnaria | Diptera |  |  |
| C |  |  | Sarcophaga melanura | Diptera |  |  |
| C |  |  | Sarcophaga sensu lato | Diptera |  |  |
| C |  |  | Sarcotachinella sinuata | Diptera |  |  |
| C |  |  | Scathophaga stercoraria | Diptera |  |  |
| C |  |  | Siphona urbana | Diptera |  |  |
| C |  |  | Sphaerophoria interrupta | Diptera |  |  |
| C |  |  | Tenthredo arcuata | Hymenoptera |  |  |
| G | Acanthiophilus helianthi | Diptera |  |  | Adia cinerella | Diptera |
|  | Adia cinerella | Diptera | Adia cinerella | Diptera | Andrena nigroaenea | Hymenoptera |
| G | Anthomyia liturata | Diptera | Anthomyia liturata | Diptera | Anthomyia liturata | Diptera |
| G | Apis mellifera | Hymenoptera | Apis mellifera | Hymenoptera | Bellardia pandia | Diptera |
| G | Azelia zetterstedti | Diptera | Bellardia pandia | Diptera | Bellardia vulgaris | Diptera |
| G | Bellardia pandia | Diptera | Bellardia vulgaris | Diptera | Bombus terrestris | Hymenoptera |
| G | Bellardia vulgaris | Diptera | Bombus lapidarius | Hymenoptera | Botanophila striolata | Diptera |
| G | Bombus lapidarius | Hymenoptera | Bombus lucorum | Hymenoptera | Cheilosia albitarsis | Diptera |
| G | Bombus lucorum | Hymenoptera | Bombus pratorum | Hymenoptera | Chloromyia formosa | Diptera |
| G | Bombus pascuorum | Hymenoptera | Botanophila striolata | Diptera | Chromatomyia nigra | Diptera |
| G | Bombus pratorum | Hymenoptera | Braconidae sp. | Hymenoptera | Coccidula rufa | Coleoptera |
| G | Botanophila striolata | Diptera | Calliphora vicina | Diptera | Coccinella septempunctata | Coleoptera |
| G | Cetema paramyopina | Diptera | Cantharis lateralis | Coleoptera | Coenosia tigrina | Diptera |
| G | Cheilosia latifrons | Diptera | Cantharis nigra | Coleoptera | Delia florilega | Diptera |
| G | Cheilosia vernalis | Diptera | Cetema neglecta | Diptera | Dilophus febrilis | Diptera |
| G | Chloromyia formosa | Diptera | Cetema paramyopina | Diptera | Dolichopus simplex | Diptera |
| G | Chrysotus sp. | Diptera | Cheilosia albitarsis | Diptera | Empis bicuspidata | Diptera |
| G | Coccinella septempunctata | Coleoptera | Chloromyia formosa | Diptera | Episyrphus balteatus | Diptera |
| G | Coenosia tigrina | Diptera | Chrysotus sp. | Diptera | Eupeodes corollae | Diptera |
| G | Coproica ferruginea | Diptera | Coccidula rufa | Coleoptera | Haematopota pluvialis | Diptera |
| G | Delia platura | Diptera | Coccinella septempunctata | Coleoptera | Helina communis | Diptera |
| G | Dilophus febrilis | Diptera | Coenosia tigrina | Diptera | Helina duplicata | Diptera |
| G | Diplonevra funebris | Diptera | Copromyza similis | Diptera | Helina obscurata | Diptera |
| G | Dolichopus caligatus | Diptera | Delia platura | Diptera | Helina parcepilosa | Diptera |
| G | Dolichopus plumipes | Diptera | Dilophus febrilis | Diptera | Hydrotaea albipuncta | Diptera |
| G | Empis albinervis | Diptera | Diplonevra funebris | Diptera | Hydrotaea cinerea | Diptera |
| G | Empis bicuspidata | Diptera | Dolichopus plumipes | Diptera | Hylemya vagans | Diptera |
| G | Episyrphus balteatus | Diptera | Empis albinervis | Diptera | Hylemya variata | Diptera |
| G | Eriothrix rufomaculata | Diptera | Empis bicuspidata | Diptera | Lonchoptera furcata | Diptera |
| G | Eristalis arbustorum | Diptera | Empis livida | Diptera | Lotophila atra | Diptera |
| G | Eristalis tenax | Diptera | Episyrphus balteatus | Diptera | Melanostoma mellinum | Diptera |
| G | Eupeodes corollae | Diptera | Eriothrix rufomaculata | Diptera | Melanostoma scalare | Diptera |
| G | Gastrophysa viridula | Coleoptera | Eupeodes corollae | Diptera | Meligethes sp. | Coleoptera |
| G | Geomyza tripunctata | Diptera | Eupeodes latifasciatus | Diptera | Meliscaeva auricollis | Diptera |
| G | Glyphipterix simpliciella | Lepidoptera | Gastrophysa viridula | Coleoptera | Muscina assimilis | Diptera |
| G | Helophilus hybridus | Diptera | Geomyza tripunctata | Diptera | Neomyia cornicina | Diptera |
| G | Helophilus pendulus | Diptera | Haematopota pluvialis | Diptera | Oedemera nobilis | Coleoptera |
| G | Hylemya variata | Diptera | Helina duplicata | Diptera | Oscinella nitidissima | Diptera |
| G | Ichneumonidae sp. | Hymenoptera | Helina obscurata | Diptera | Pegoplata aestiva | Diptera |
| G | Lonchoptera furcata | Diptera | Hilara sp. | Diptera | Phaonia incana | Diptera |
| G | Lonchoptera lutea | Diptera | Hydrellia sp. | Diptera | Phaonia tuguriorum | Diptera |
| G | Lotophila atra | Diptera | Hylemya vagans | Diptera | Platycheirus albimanus | Diptera |
| G | Malachius bipustulatus | Coleoptera | Hylemya variata | Diptera | Platycheirus clypeatus | Diptera |
| G | Maniola jurtina | Lepidoptera | Ischnopterapion loti | Coleoptera | Platycheirus granditarsus | Diptera |
| G | Melanostoma mellinum | Diptera | Lejogaster metallina | Diptera | Pollenia labialis | Diptera |
| G | Melanostoma scalare | Diptera | Lonchoptera lutea | Diptera | Pollenia pediculata | Diptera |
| G | Meligethes sp. | Coleoptera | Lotophila atra | Diptera | Pollenia rudis | Diptera |
| G | Micropterix calthella | Lepidoptera | Megaselia sp. | Diptera | Protapion fulvipes | Coleoptera |
| G | Myospila meditabunda | Diptera | Melanostoma mellinum | Diptera | Pyralidae sp. | Lepidoptera |
| G | Neomyia cornicina | Diptera | Melanostoma scalare | Diptera | Rhagonycha fulva | Coleoptera |
| G | Neomyia viridescens | Diptera | Meligethes sp. | Coleoptera | Saltella sphondylii | Diptera |
| G | Oedemera lurida | Coleoptera | Metopina sp. | Diptera | Sarcophaga sensu lato | Diptera |
| G | Opomyza germinationis | Diptera | Micropterix calthella | Lepidoptera | Scathophaga stercoraria | Diptera |
| G | Pegoplata aestiva | Diptera | Myospila meditabunda | Diptera | Siphona urbana | Diptera |
| G | Platycheirus albimanus | Diptera | Neomyia cornicina | Diptera | Sphaerophoria interrupta | Diptera |
| G | Platycheirus clypeatus | Diptera | Opomyza germinationis | Diptera | Sphaerophoria scripta | Diptera |
| G | Platycheirus granditarsus | Diptera | Oscinella frit | Diptera | Staphylinidae sp. | Coleoptera |
| G | Platycheirus manicatus | Diptera | Pegoplata aestiva | Diptera | Tenthredo arcuata | Hymenoptera |
| G | Pollenia amentaria | Diptera | Platycheirus albimanus | Diptera | Tipula paludosa | Diptera |
| G | Pollenia pediculata | Diptera | Platycheirus clypeatus | Diptera |  |  |
| G | Pollenia rudis | Diptera | Platycheirus europaeus | Diptera |  |  |
| G | Protapion apricans | Coleoptera | Pollenia rudis | Diptera |  |  |
| G | Protapion fulvipes | Coleoptera | Protapion apricans | Coleoptera |  |  |
| G | Protapion trifolii | Coleoptera | Protapion fulvipes | Coleoptera |  |  |
| G | Pyralidae sp. | Lepidoptera | Protapion trifolii | Coleoptera |  |  |
| G | Pyronia tithonus | Lepidoptera | Pyralidae sp. | Lepidoptera |  |  |
| G | Rhagonycha fulva | Coleoptera | Rhagio scolapaceus | Diptera |  |  |
| G | Rhingia campestris | Diptera | Rhagonycha fulva | Coleoptera |  |  |
| G | Saltella sphondylii | Diptera | Rhingia campestris | Diptera |  |  |
| G | Sarcophaga melanura | Diptera | Saltella sphondylii | Diptera |  |  |
| G | Sarcophaga sensu lato | Diptera | Sarcophaga carnaria | Diptera |  |  |
| G | Scaeva pyrastri | Diptera | Sarcophaga sensu lato | Diptera |  |  |
| G | Scathophaga stercoraria | Diptera | Sarcophaga subvicina | Diptera |  |  |
| G | Siphona urbana | Diptera | Scathophaga stercoraria | Diptera |  |  |
| G | Spelobia sp. | Diptera | Siphona urbana | Diptera |  |  |
| G | Sphaerophoria interrupta | Diptera | Sphaerophoria interrupta | Diptera |  |  |
| G | Syritta pipiens | Diptera | Tachinus sp. | Coleoptera |  |  |
| G | Syrphus ribesii | Diptera | Tenthredo arcuata | Hymenoptera |  |  |
| G | Tenthredo arcuata | Hymenoptera |  |  |  |  |
|  |  |  |  |  |  |  |
|  |  |  |  |  |  |  |
|  |  |  |  |  |  |  |
|  |  |  |  |  |  |  |
|  |  |  |  |  |  |  |
|  |  |  |  | |  |  |
|  |  |  |  | |  |  |
|  |  |  |  | |  |  |

Table S6. Species lists of pollinators found on each farm in the 2012 and 2013 surveys.

| **Farm 1** | |
| --- | --- |
| **Pollinator species** | **Pollinator order** |
| Adia cinerella | Diptera |
| Bombus terrestris | Hymenoptera |
| Botanophila striolata | Diptera |
| Braconidae sp. | Hymenoptera |
| Chaetorellia jaceae | Diptera |
| Cheilosia albitarsis | Diptera |
| Delia platura | Diptera |
| Empis albinervis | Diptera |
| Eristalis arbustorum | Diptera |
| Fannia fuscula | Diptera |
| Fannia serena | Diptera |
| Helina parcepilosa | Diptera |
| Hydrellia griseola | Diptera |
| Hylemya variata | Diptera |
| Lasioglossum calceatum | Hymenoptera |
| Lasius niger | Hymenoptera |
| Lotophila atra | Diptera |
| Melanostoma mellinum | Diptera |
| Meligethes sp. | Coleoptera |
| Neomyia cornicina | Diptera |
| Oedemera nobilis | Coleoptera |
| Oscinella frit | Diptera |
| Pegoplata aestiva | Diptera |
| Pegoplata debilis | Diptera |
| Pegoplata infirma | Diptera |
| Phyllobius pyri | Coleoptera |
| Platycheirus albimanus | Diptera |
| Platycheirus granditarsus | Diptera |
| Pollenia amentaria | Diptera |
| Protapion trifolii | Coleoptera |
| Rhagonycha fulva | Coleoptera |
| Rhamphomyia longipes | Diptera |
| Scathophaga stercoraria | Diptera |
| Sciara hemerobioides | Diptera |
| Spelobia sp. | Diptera |
| Syritta pipiens | Diptera |

| **Farm 2** | |
| --- | --- |
| **Pollinator species** | **Pollinator order** |
| Adia cinerella | Diptera |
| Andrena haemorrhoa | Hymenoptera |
| Anthomyia liturata | Diptera |
| Apis melifera | Hymenoptera |
| Bicellaria vana | Diptera |
| Bombus pascuorum | Hymenoptera |
| Botanophila striolata | Diptera |
| Braconidae sp. | Hymenoptera |
| Chalcodoidea sp. | Hymenoptera |
| Cheilosia albitarsis | Diptera |
| Cheilosia latifrons | Diptera |
| Cheilosia pagana | Diptera |
| Chironomidae sp. | Diptera |
| Copromyza equina | Diptera |
| Delia platura | Diptera |
| Dolichopus plumipes | Diptera |
| Empididae sp. | Diptera |
| Empis caudatula | Diptera |
| Empis opaca | Diptera |
| Episyrphus balteatus | Diptera |
| Eristalis arbustorum | Diptera |
| Eucoilidae sp. | Hymenoptera |
| Eudasyphora cyanicolor | Diptera |
| Fannia mollissima | Diptera |
| Halictus tumulorum | Hymenoptera |
| Hydrellia griseola | Diptera |
| Lasioglossum calceatum | Hymenoptera |
| Lasioglossum puncticolle | Hymenoptera |
| Lonchoptera furcata | Diptera |
| Lotophila atra | Diptera |
| Melanostoma mellinum | Diptera |
| Melanostoma scalare | Diptera |
| Meligethes sp. | Coleoptera |
| Neoascia podagrica | Diptera |
| Oedemera nobilis | Coleoptera |
| Orthonevra nobilis | Diptera |
| Oscinella frit | Diptera |
| Pegoplata aestiva | Diptera |
| Pegoplata debilis | Diptera |
| Phytomyza vitalbae | Diptera |
| Phytomyza wahlgreni | Diptera |
| Platycheirus albimanus | Diptera |
| Protapion trifolii | Coleoptera |
| Rhagonycha fulva | Coleoptera |
| Rhamphomyia longipes | Diptera |
| Saltella sphondylii | Diptera |
| Scathophaga stercoraria | Diptera |
| Schoenomyza litorella | Diptera |
| Sepsis cynipsea | Diptera |
| Siphona geniculata | Diptera |
| Sphaerophoria scripta | Diptera |
| Sympycnus desoutteri | Diptera |
| Syritta pipiens | Diptera |

| **Farm 3** | |
| --- | --- |
| **Pollinator species** | **Pollinator order** |
| Adia cinerella | Diptera |
| Andrena wilkella | Hymenoptera |
| Apis melifera | Hymenoptera |
| Bombus lucorum | Hymenoptera |
| Bombus pascuorum | Hymenoptera |
| Bombus pratorum | Hymenoptera |
| Bombus terrestris | Hymenoptera |
| Botanophila striolata | Coleoptera |
| Braconidae sp. | Hymenoptera |
| Chalcodoidea sp. | Hymenoptera |
| Chironomidae sp. | Diptera |
| Chloromyia formosa | Diptera |
| Coccinella septempunctata | Coleoptera |
| Delia sp. | Diptera |
| Episyrphus balteatus | Diptera |
| Eristalis arbustorum | Diptera |
| Eupeodes luniger | Diptera |
| Harmonia axyridis | Coleoptera |
| Hydrellia griseola | Diptera |
| Lasius niger | Hymenoptera |
| Malachius bipustulatus | Coleoptera |
| Melanostoma mellinum | Diptera |
| Meligethes sp. | Coleoptera |
| Oedemera nobilis | Coleoptera |
| Oscinella frit | Diptera |
| Oscinella nigerrima | Diptera |
| Pegoplata aestiva | Diptera |
| Phytomyza wahlgreni | Diptera |
| Protapion fulvipes | Coleoptera |
| Protapion trifolii | Coleoptera |
| Saltella sphondylii | Diptera |
| Scathophaga stercoraria | Diptera |
| Sepsis fulgens | Diptera |
| Tipulidae sp. | Diptera |

| **Farm 4** | |
| --- | --- |
| **Pollinator species** | **Pollinator order** |
| Braconidae sp. | Hymenoptera |
| Cecidomyiidae sp. | Diptera |
| Chironomidae sp. | Diptera |
| Delia platura | Diptera |
| Dilophus febrilis | Diptera |
| Eristalis arbustorum | Diptera |
| Eucoilidae sp. | Hymenoptera |
| Geomyza tripunctata | Diptera |
| Hybomitra micans | Diptera |
| Hydrellia griseola | Diptera |
| Hydrellia sp. | Diptera |
| Lonchoptera bifurcata | Diptera |
| Lotophila atra | Diptera |
| Megaselia sp. | Diptera |
| Melanostoma mellinum | Diptera |
| Meligethes sp. | Coleoptera |
| Oscinella nitidissima | Diptera |
| Pegoplata aestiva | Diptera |
| Platycheirus granditarsus | Diptera |
| Protapion fulvipes | Coleoptera |
| Protapion trifolii | Coleoptera |
| Rhamphomyia sulcata | Diptera |
| Scathophaga stercoraria | Diptera |
| Sciara hemerobioides | Diptera |
| Sepsis cynipsea | Diptera |
| Tipulidae sp. | Diptera |

| **Farm 5** | |
| --- | --- |
| **Pollinator species** | **Pollinator order** |
| Amara similata | Coleoptera |
| Bicellaria vana | Diptera |
| Chalcidoidea sp. | Hymenoptera |
| Delia sp. | Diptera |
| Hydrellia sp. | Diptera |
| Hylemya variata | Diptera |
| Lonchoptera bifurcata | Diptera |
| Lonchoptera lutea | Diptera |
| Melanostoma mellinum | Diptera |
| Meligethes sp. | Coleoptera |
| Meoneura sp. | Diptera |
| Phora sp. | Diptera |
| Protapion fulvipes | Coleoptera |
| Saltella sphondylii | Diptera |
| Scathophaga stercoraria | Diptera |
| Sepsis cynipsea | Diptera |

| **Farm 6** | |
| --- | --- |
| **Pollinator species** | **Pollinator order** |
| Andrena angustior | Hymenoptera |
| Andrena cineraria | Hymenoptera |
| Bicellaria vana | Diptera |
| Bombus lapidarius | Hymenoptera |
| Bombus terrestris | Hymenoptera |
| Botanophila fugax | Diptera |
| Botanophila striolata | Diptera |
| Brontaea humilis | Diptera |
| Cerodontha denticornis | Diptera |
| Ceutorhynchus erysimi | Coleoptera |
| Ceutorhynchus obstrictus | Coleoptera |
| Cheilosia albitarsis | Diptera |
| Chloromyia formosa | Diptera |
| Chrysotus monochaetus | Diptera |
| Chrysotus sp. | Diptera |
| Copromyza equina | Diptera |
| Copromyza similis | Diptera |
| Delia platura | Diptera |
| Dolichopus agilis | Diptera |
| Empis opaca | Diptera |
| Episyrphus balteatus | Diptera |
| Eristalis tenax | Diptera |
| Fannia serena | Diptera |
| Harmonia axyridis | Coleoptera |
| Hydrellia griseola | Diptera |
| Hylemya vagans | Diptera |
| Lotophila atra | Diptera |
| Melanostoma mellinum | Diptera |
| Meligethes sp. | Coleoptera |
| Neoascia podagrica | Diptera |
| Pegoplata aestiva | Diptera |
| Pegoplata infirma | Diptera |
| Pipiza bimaculata | Diptera |
| Platycheirus albimanus | Diptera |
| Propylea quatuordecimpunctata | Coleoptera |
| Rhagonycha fulva | Coleoptera |
| Saltella sphondylii | Diptera |
| Scathophaga stercoraria | Diptera |
| Sepsis cynipsea | Diptera |
| Sympycnus desoutteri | Diptera |
| Themira annulipes | Diptera |

| **Farm 7** | |
| --- | --- |
| **Pollinator species** | **Pollinator order** |
| Anthomyia liturata | Diptera |
| Athous bicolor | Coleoptera |
| Bombus lapidarius | Hymenoptera |
| Botanophila striolata | Diptera |
| Delia platura | Diptera |
| Empis livida | Diptera |
| Episyrphus balteatus | Diptera |
| Fannia serena | Diptera |
| Hylemya vagans | Diptera |
| Hylemya variata | Diptera |
| Melanostoma mellinum | Diptera |
| Meligethes sp. | Coleoptera |
| Phora sp. | Diptera |
| Platycheirus albimanus | Diptera |
| Protapion fulvipes | Coleoptera |
| Reichertella geniculata | Diptera |
| Scathophaga stercoraria | Diptera |
| Siphona geniculata | Diptera |

| **Farm 8** | |
| --- | --- |
| **Pollinator species** | **Pollinator order** |
| Adia cinerella | Diptera |
| Agriotes sputator | Coleoptera |
| Andrena wilkella | Hymenoptera |
| Apis mellifera | Hymenoptera |
| Bicellaria vana | Diptera |
| Bombus hortorum | Hymenoptera |
| Bombus lapidarius | Hymenoptera |
| Bombus lucorum | Hymenoptera |
| Bombus pascuorum | Hymenoptera |
| Bombus pratorum | Hymenoptera |
| Bombus terrestris | Hymenoptera |
| Bombus vestalis | Hymenoptera |
| Botanophila fugax | Diptera |
| Botanophila striolata | Diptera |
| Cantharis rustica | Coleoptera |
| Cheilosia pagana | Diptera |
| Delia platura | Diptera |
| Empis caudatula | Diptera |
| Empis femorata | Diptera |
| Empis livida | Diptera |
| Episyrphus balteatus | Diptera |
| Eristalis arbustorum | Diptera |
| Eristalis tenax | Diptera |
| Glyphipterix simpliciella | Lepidoptera |
| Halictus tumulorum | Hymenoptera |
| Helophilus pendulus | Diptera |
| Hilara sp. | Diptera |
| Hylemya variata | Diptera |
| Hypera meles | Coleoptera |
| Lasioglossum albipes | Hymenoptera |
| Lasioglossum calceatum | Hymenoptera |
| Lasius niger | Hymenoptera |
| Lepidoptera | Lepidoptera |
| Limnia unguicornis | Diptera |
| Lonchoptera bifurcata | Diptera |
| Lotophila atra | Diptera |
| Melanargia galathea | Lepidoptera |
| Melanostoma mellinum | Diptera |
| Melanostoma scalare | Diptera |
| Meligethes sp. | Coleoptera |
| Melitta leporina | Hymenoptera |
| Merodon equestris | Hymenoptera |
| Mydaea anicula | Diptera |
| Myospila meditabunda | Diptera |
| Oedemera lurida | Coleoptera |
| Oscinella frit | Diptera |
| Pegoplata aestiva | Diptera |
| Pherbellia cinerella | Diptera |
| Pieris brassicae | Lepidoptera |
| Platycheirus albimanus | Diptera |
| Protapion abricans | Coleoptera |
| Protapion fulvipes | Coleoptera |
| Protapion trifolii | Coleoptera |
| Rhagonycha fulva | Coleoptera |
| Scathophaga stercoraria | Diptera |
| Sciara hemerobioides | Diptera |
| Sepsis fulgens | Diptera |
| Siphona geniculata | Diptera |
| Sphaerophoria scripta | Diptera |

| **Farm 9** | |
| --- | --- |
| **Pollinator species** | **Pollinator order** |
| Andrena cineraria | Hymenoptera |
| Andrena dorsata | Hymenoptera |
| Andrena nitida | Hymenoptera |
| Aphthona euphorbiae | Coleoptera |
| Botanophila striolata | Diptera |
| Braconidae sp. | Hymenoptera |
| Chalcidoidea sp. | Hymenoptera |
| Cheilosia albitarsis | Diptera |
| Chironomidae sp. | Diptera |
| Coccinella septempunctata | Coleoptera |
| Delia sp. | Diptera |
| Dilophus febrilis | Diptera |
| Empis femorata | Diptera |
| Empis aemula | Diptera |
| Eriothrix rufomaculata | Diptera |
| Eristalinus sepulchralis | Diptera |
| Eristalis interruptus | Diptera |
| Fannia serena | Diptera |
| Harmonia axyridis | Coleoptera |
| Helina setiventris | Diptera |
| Helophilus trivittatus | Diptera |
| Hydrellia griseola | Diptera |
| Lonchoptera bifurcata | Diptera |
| Lotophila atra | Diptera |
| Malachius bipustulatus | Coleoptera |
| Melanostoma mellinum | Diptera |
| Melanostoma scalare | Diptera |
| Meligethes aeneus | Coleoptera |
| Melinda gentilis | Diptera |
| Merodon equestris | Hymenoptera |
| Microcercis albipalpis | Diptera |
| Neomyia viridescens | Diptera |
| Oedemera nobilis | Coleoptera |
| Oscinella frit | Diptera |
| Pegoplata aestiva | Diptera |
| Phyllobius pyri | Coleoptera |
| Phytomyza notata | Diptera |
| Platycheirus albimanus | Diptera |
| Rhagonycha fulva | Coleoptera |
| Sarcophaga carnaria | Diptera |
| Sarcophaga variegata | Diptera |
| Scathophaga stercoraria | Diptera |
| Sciara hemerobioides | Diptera |
| Sepsis cynipsea | Hymenoptera |
| Syritta pipiens | Diptera |
| Themira annulipes | Diptera |

| **Farm 10** | |
| --- | --- |
| **Pollinator species** | **Pollinator order** |
| Adia cinerella | Diptera |
| Braconidae sp. | Hymenoptera |
| Chironomidae sp. | Coleoptera |
| Delia platura | Diptera |
| Hydrellia sp. | Diptera |
| Lotophila atra | Diptera |
| Meligethes sp. | Coleoptera |
| Rhamphomyia sulcata | Diptera |
| Scathophaga stercoraria | Diptera |

Table S7. Correlations between the parameters of the pollinator communities in both the field-experiment and farm pollinator surveys

Field-experiment correlations

|  | Pollinator species richness | Pollinator abundance |
| --- | --- | --- |
| Pollinator Functional Diversity | p<0.001  R=0.93 | p<0.001  R=0.77 |
| Pollinator species richness | - | p<0.001  R=0.82 |

Farm surveys correlations

|  | Pollinator species richness | Pollinator abundance |
| --- | --- | --- |
| Pollinator Functional Diversity | p<0.001  R=0.90 | p<0.001  R=0.80 |
| Pollinator species richness | - | P<0.001  R=0.82 |

**References**

Macfadyen, S., Gibson, R., Polaszek, A., Morris, R.J., Craze, P.G., Planque, R., Symondson, W.O.C. & Memmott, J. (2009) Do differences in food web structure between organic and conventional farms affect the ecosystem service of pest control? *Ecology Letters,* **12,** 229-238.

Rodwell, J.S. (1992) *British Plant Communities. Volume 3. Grasslands and Montane Communities.* Cambridge University Press, Cambridge, UK.
